# The contribution of cause-effect link to representing the core of scientific paper—The role of Semantic Link Network

**DOI:** 10.1371/journal.pone.0199303

**Published:** 2018-06-21

**Authors:** Mengyun Cao, Xiaoping Sun, Hai Zhuge

**Affiliations:** 1 Laboratory of Cyber-Physical-Social Intelligence, Guangzhou University, China; 2 Key Laboratory of Intelligent Information Processing at Institute of Computing Technology, University of Chinese Academy of Sciences, Chinese Academy of Sciences, Beijing, China; 3 Systems Analytics Research Institute, Aston University, Birmingham, United Kingdom; Universitat Rovira i Virgili, SPAIN

## Abstract

The Semantic Link Network is a general semantic model for modeling the structure and the evolution of complex systems. Various semantic links play different roles in rendering the semantics of complex system. One of the basic semantic links represents cause-effect relation, which plays an important role in representation and understanding. This paper verifies the role of the Semantic Link Network in representing the core of text by investigating the contribution of cause-effect link to representing the core of scientific papers. Research carries out with the following steps: (1) Two propositions on the contribution of cause-effect link in rendering the core of paper are proposed and verified through a statistical survey, which shows that the sentences on cause-effect links cover about 65% of key words within each paper on average. (2) An algorithm based on syntactic patterns is designed for automatically extracting cause-effect link from scientific papers, which recalls about 70% of manually annotated cause-effect links on average, indicating that the result adapts to the scale of data sets. (3) The effects of cause-effect link on four schemes of incorporating cause-effect link into the existing instances of the Semantic Link Network for enhancing the summarization of scientific papers are investigated. The experiments show that the quality of the summaries is significantly improved, which verifies the role of semantic links. The significance of this research lies in two aspects: (1) it verifies that the Semantic Link Network connects the important concepts to render the core of text; and, (2) it provides an evidence for realizing content services such as summarization, recommendation and question answering based on the Semantic Link Network, and it can inspire relevant research on content computing.

## Introduction

Text is a kind of representation that conveys idea. It can be classified into the following types: (1) *Description*, describing what the thing is like; (2) *Exposition*, explaining or informing things; (3) *Argument*, convincing someone to accept some opinions; and, (4) *Narration*, narrating the development of a series of events [1]. An article can be one or a combination of these types. Scientific paper usually combines these types and more emphasizes on expositions and arguments.

A meaningful text contains abundant semantic links such as *is-part-of* link and *cause-effect* link, revealing the connection between described entities, events, assertions and arguments, and integrating the meanings conveyed by different language expression units (such as word, sentence and passage) into the meaning of text. As a kind of formal and rigorous texts, scientific papers contain rich semantic links. Different semantic links play different roles in rendering different contents. The *cause-effect* link is one of the basic semantic links that takes part in rational thinking. Cognitive psychologists have shown the role of *cause-effect* link in understanding narrative texts, and shown that causal relation is decisive on identifying important events.

The Semantic Link Network is a self-organized semantic model for representing and operating the semantic structure of complex system [2, 3]. Its nodes represent categories of things and the links between nodes represent the semantic relations between nodes. A text can be transformed into an instance of Semantic Link Network where words, sentences and paragraphs are connected by semantic links such as the *is-part-of* link and cause-effect link [4–6].

The motivation of this paper is to verify the proposition that semantic links play an important role in representing the core of text [6]. Research carries out with investigating the role of *cause-effect* link in representing the core of scientific papers. A better understanding of how the *cause-effect* link contributes to the core of text can inspire research on content services such as automatic summarization, recommendation and question answering.

This research focuses on analyzing the clause-level *cause-effect* link (either the cause component or the effect component contains at least one clause) since the *cause-effect* link at noun-phrase-level [7] and event-level [8] is more suitable for analyzing narrative text. For simplicity, this paper uses *A* → *B* to represent that *A* is the cause of *B*.

Research carries out with the following three steps.

1. *Observation*. We invited professionals to manually annotate clause-level *cause-effect* links within a set of given papers, and observed the distribution of *cause-effect* links and the coverage of words on *cause-effect* links within each paper. Two propositions are proposed: a) *the distribution of cause-effect links indicates the intensity of representation;* and, b) *cause-effect links cover the key words within paper*. The high coverage of key words indicates that the *cause-effect* link connects important concepts.
2. *Automatic discovery of cause-effect link*. We designed an algorithm based on syntactic pattern matching to automatically extract *cause-effect* links from more papers, and conducted experiments to show that the algorithm gets a high recall and can extract *cause-effect* links missed from manual annotation. These automatically extracted *cause-effect* links are not only used to support the proposed propositions but also used to generate summaries for papers, which provides further verification through checking what kinds of semantic links are contained in summaries.
3. *Comparison*. We extracted several instances of Semantic Link Network with *is-part-of* link and *similarity* link from each scientific paper, designed four schemes of incorporating *cause-effect* links into the existing semantic link networks, and applied ranking operations on the original semantic link networks within text and the one incorporating cause-effect link to automatically generate the summaries for each paper. The common standards for summarization are used to evaluate the results for observing whether the quality of the automatically generated summaries are improved or not after incorporating the *cause-effect* link into the existing semantic link network, reflecting the role of *cause-effect* link in organizing and representing the content of scientific papers.

## Related works

Relevant research on Semantic Link Network can be traced to the discovery of the rules of inheritance in object-oriented environment in 1998 and Active Document Framework in 2003 respectively [9, 10]. The Semantic Link Network was used to organize a semantic space and effectively operate Web resources [11, 12]. It has developed a systematic theory and method for representing the basic semantic structure of various complex systems [4]. The theory and method of the Semantic Link Network have been applied to various application areas [2, 6, 13–17].

Different from the traditional Semantic Net, Semantic Link Network emphasizes on an open “Link”, on the basic operation of self-organizing a complex system just as the hyperlink self-organizing the World Wide Web, on the emerging semantics [2], and on the automatic discovery of semantic links.

The relevant initiatives include Tim Berners-Lee’s Linked Data proposed in 2006 for publishing structured data so that data can be interlinked and become more useful through semantic queries [18], and Google’s Knowledge Graph proposed in 2012 for structuring resources gathered from a wide variety of sources and search results [19]. Compared with these initiatives, the Semantic Link Network has the following distinguished characteristics: social network, dynamicity, rules, openness, self-organization, complex reasoning, order sensitive, support basic intelligence, locality and global influence, and multiple spaces introduced in [4]. Integrating Semantic Link Network with a multi-dimensional Resource Space Model forms a complex semantic model [12, 20], which has been developed toward a basic mechanism for constructing the Cyber-Physical Society [4, 6, 17, 20].

Researchers from different domains hold different views towards the specific definition of causality, e.g., “what do *A* and *B* exactly refer to” and “under what conditions we deem there exists a cause-effect relation between *A* and *B*”.

In philosophy, Aristotle discussed the causes of the existence and the change of things, and proposed the doctrine of *Four Causes* [21]. David Hume viewed causality as a kind of association between two states or occurrences that is concluded by observation [22]. The concept of probabilistic causality was also proposed [23].

In psychology, the *attribution theory* is proposed to analyze the causality that exists among people’s activities [24, 25]. The term “attribution” refers to the causal interpretation and inference conducted by observers to learn the motivation of behaviors. Cause-effect relation discussed in *attribution theory* is mainly about the correlation between behavior and motivation, belief and the external environment.

The role of cause-effect relation within texts has drawn much attention from scholars in the field of cognition, psychology and pedagogy since 1980s. Lots of studies focused on narrative texts, showing that identifying and automatically inferring cause-effect relations among events described in the texts has a crucial impact on the comprehension process of readers. The influences are mainly on three aspects: (1) *Reading speed*. The stronger the causal relatedness leads to less reading time [26, 27]. (2) *Recalling content*. The events that have more quantity and higher quality of causal relatedness with other events are quickly recalled by readers [28–30], and the events that are closer to the mainline are easier to recall [31]. (3) *Importance of event*. Causality plays a decisive role in identifying important events, and events with higher importance are more likely to be used by readers for generating abstracts [31, 32]. However, few works have been down on studying the role of cause-effect link within other types of texts, especially the complex text like scientific paper.

How to automatically extract cause-effect relation from natural language text and appropriately apply automatically extracted cause-effect relation to various application tasks has been an important issue in natural language processing and computational linguistics. The methods for automatically extracting cause-effect relations from natural language texts can be broadly divided into the following two types [33]:

1. *Pattern matching methods* [34–37]. This type of methods usually needs manual design and mainly consider lexical, syntactic and semantic features. The types of texts (such as narration, exposition, etc.) and their fields (such as financial news, stories, etc.) influence the performance of the methods because most designs of cause-effect patterns depend on domain knowledge.
2. *Statistics and machine learning methods* [38–40]. This type of methods can handle large-scale datasets and has better scalability. However, most of them require large amounts of annotated corpora, and their performances on extracting cause-effect relation between complex events and larger language units (such as sentences and paragraphs) are less effective.

Cause-effect relations automatically extracted from corpora can be applied to many application tasks of natural language processing such as automatic summarization [6], question-answering [41–44], information retrieval [45], and event prediction [46]. Cause-effect relation is inextricably bound up with many semantic relations: (1) the judgment process of causality involves temporal relation, conditional relation and hierarchical relation [47]; and, (2) some semantic relations such as temporal relation, condition, material, usage, reason, goal, and prevention can be considered as describing cause-effect relation from different perspectives [48, 49]. Therefore, we should carefully choose the expression model of cause-effect relation for different applications.

## General architecture of research

Research on Semantic Link Network has built a systematic theory and method for representing the fundamental structure of complex systems, especially the self-organizing complex systems [4, 6, 16, 17]. Previous works have shown that Semantic Link Network can effectively organize and express the core of some complex systems [2, 6, 12].

This research focuses on the role of *cause-effect* link in scientific papers through observation, automatic extraction and automatic summarization. Verifying the role of *cause-effect* link in scientific papers provides the evidence for the significance of Semantic Link Network in expressing the core of text.

**Fig 1:**
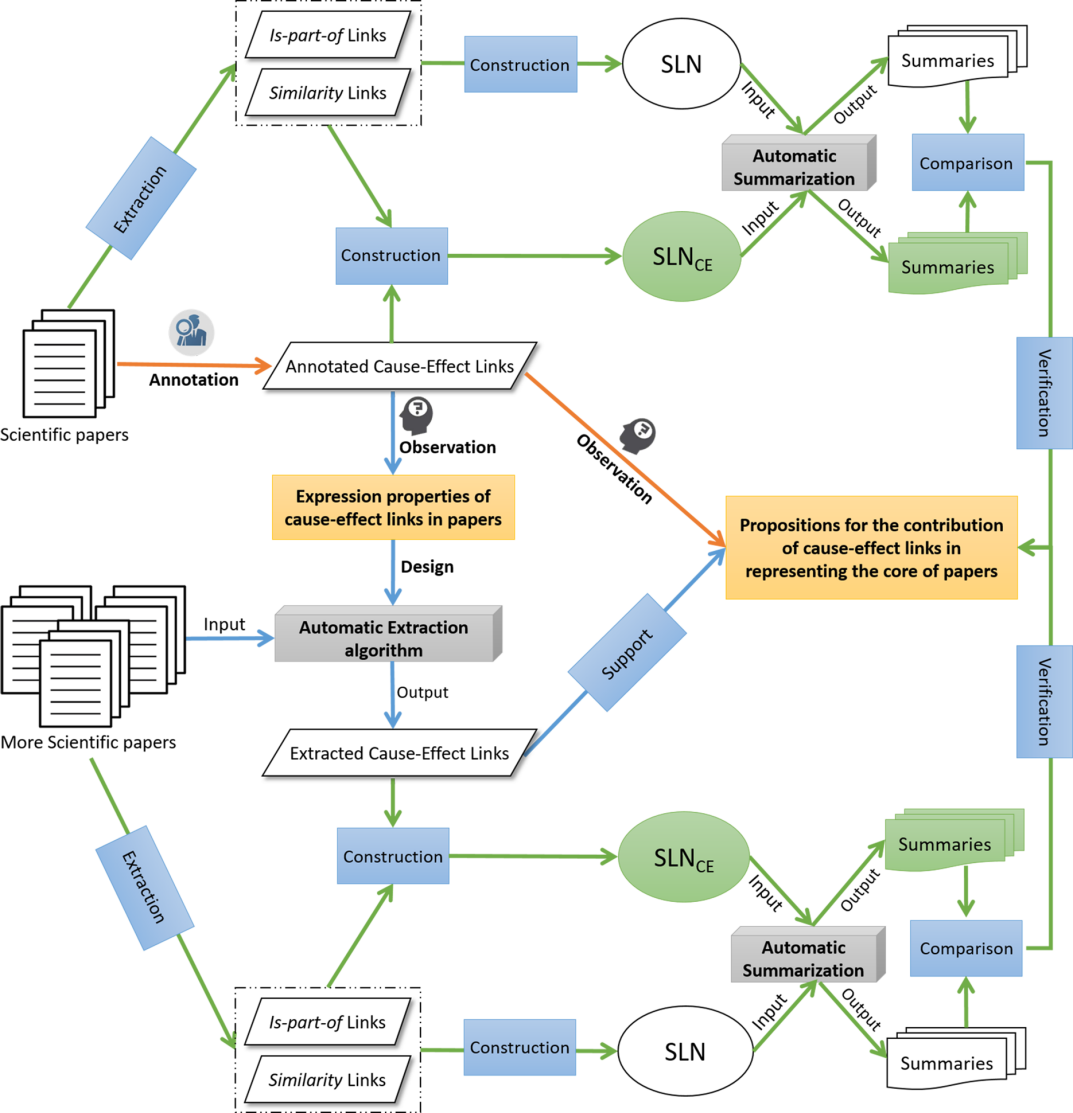
The general architecture of this research.

Fig 1 depicts the general architecture of this study, where the arrows in orange color connect two components of the first step of the experiment, the arrows in blue color connect the first step to the second step, and the arrows in green color connect the second step to the third step. The rectangles in orange denote the important propositions proposed in our experiment. The white ellipse with the label *“SLN”* represents an instance of Semantic Link Network that does not contain *cause-effect* links, while the green ellipse with the label *“SLN*_*CE*_*”* represents the enhanced instance that combines the manually annotated or automatically extracted *cause-effect* links. A parallelogram refers to a collection of semantic links, a blue rectangle represents a procedure to process or analyze data, and a grey cuboid represents an algorithm.

## Propositions

Observational experiment starts from selecting 39 scientific papers of three researchers in computer science (13 papers were selected for each author), and inviting annotators majoring in computer science to annotate *cause-effect* links within 9 papers of these papers (3 papers for each author). These 39 papers are named *EMY* (Expanded *MY*) dataset, and the 9 annotated papers within the *EMY* dataset are named *OBSERVATION* dataset. Table 1 lists the details of our experimental datasets. Grouping papers by authors makes it easier for observing causal cues (words or phrases such as “*because*” and “*due to*” indicate *cause-effect* links). The purpose is to implement a pattern-based algorithm for automatically extracting *cause-effect* links.

**Table 1:** The experimental datasets used in this paper and in our previous work.

Dataset Name	Brief Description	Paper Number & ID	Annotated Paper Number & ID	Annotations
*MY*	The journal paper dataset used in [50]	13 (f0001-f0013)	3 (f0001-f0003)	*Cause-effect* links
*EMY*	The expanded journal paper dataset	39 (f0001-f0039)	9 (f0001-f0003, f0014-f0016, f0027-f0029)	1. *Cause-effect* links 2. Manual summaries for each section
*OBSERVATION*	Annotated papers within the *EMY* dataset	9 (f0001-f0003, f0014-f0016, f0027-f0029)	9 (f0001-f0003, f0014-f0016, f0027-f0029)	1. *Cause-effect* links 2. Manual summaries for each section
*ACL2014*	The conference paper dataset collected from proceedings of ACL 2014	173	0	(No manually annotation)

Three principles are introduced in S2 Appendix for helping the annotators to judge whether text snippet *A* is the cause of the text snippet *B*. Table 2 lists the quantity of the annotated *cause-effect* links within each paper of the *OBSERVATION* dataset, where *S_num* denotes the number of sentences within each paper, *CE_num* denotes the number of annotated *cause-effect* links, and *CE_rate* equals to *S_num/CE_num*.

**Table 2:** The intensity of annotated cause-*effect* links within each paper of the *OBSERVATION* dataset.

Article ID	S_num	CE_num	CE_rate
f0001	712	92	7.74
f0002	167	28	5.96
f0003	1106	145	7.63
f0014	102	36	2.83
f0015	194	34	5.71
f0016	162	35	4.63
f0027	96	23	4.17
f0028	279	48	5.81
f0029	241	36	6.69
**Average**	339.89	53	5.69

Table 2 shows that *CE_num* is in proportion to *S_num* (i.e., the more sentences a paper contains, the more *cause-effect* links are labeled), and the average *CE_rate* is 5.69, which indicates that every 6 sentences contain a *cause-effect* link. This indicates that *cause-effect* links are abundant within scientific paper.

The distribution of *cause-effect* links on sections and the key word coverage of *cause-effect* links indicate the following two propositions.

**Proposition 1. The Distribution of Cause-Effect Link Indicates the Intensity of Representation**.

This can be observed in Table 3, which shows the distribution of *cause-effect* links annotated within paper f0001. The coverage rate of sentences, denoted as *Cover Rate*, is the percentage of sentences containing *cause-effect* links to the total number of sentences within a section. The *Cover Rate* measures the intensity of sentences involved *cause-effect* links within a section. A section with a high *Cover Rate* indicates that many sentences within this section are used to express *cause-effect* links, thus having a higher intensity of descriptions of *cause-effect* links or a higher intensity for the descriptions of cause-effect reasoning procedure. That is, one *cause-effect* link can involve many sentences to represent itself or to render a logical reasoning process. For example, in section 3 of paper f0001, the author introduces a major *cause-effect* link where the segment about *“basic characteristics and principles of language use and understanding”* is treated as the cause and the segment about *“strategies for summarization”* is deemed as the effect. That is, the whole section mainly describes one *cause-effect* link with the first half of section on the cause and the second half of section on the effect. Thus, this section has a very high *Cover Rate* of 98% (See Table 3). Therefore, *Cover Rate* reflects the intensity of representations of *cause-effect* links within a section.

**Table 3:** The distribution of *cause-effect* link on the sections of paper f0001.

Section Title	Sentence Number	Cover Rate (%)
Abstract	12	25
1. Introduction	118	9.32
2. Multi-Dimensional Methodology	36	5.56
3. Basic Characteristics and Principles …	70	98.60
4. General Citation–Definition …	81	27.16
5. Dimension of Representation	99	14.14
6. Multi-Dimensional Evaluation	38	23.68
7. Incorporating pictures into summary	77	25.97
8. Summarizing Videos, Graphs and Pictures	75	21.33
9. General Summarization	93	21.51
10. Conclusion	13	7.70

Table 3 shows that the coverage rates of sentences on some sections are much higher than others. For examples, section 3 *“Basic Characteristics and Principles…”* has a *Cover Rate* exceeding 98%, section 4 *“General Citation…”* has a relatively higher *Cover Rate* than section *“Introduction”* and section 2 *“Multi-Dimensional Methodology”*. This indicates that sections with higher *Cover Rate* reflect the higher intensity of representing *cause-effect* links.

The distributions of the annotated *cause-effect* links within each paper of the *OBSERVATION* dataset are given in S4 Appendix, confirming that sections with higher *Cover Rate* reflect the higher intensity of representing *cause-effect* links.

*Cause-effect* links enable authors to present their ideas logically, which enables readers to convince representation and facilitate their understanding. For narrative texts, cause-effect relations contribute to rendering the importance of events [31], and events with higher importance are more likely to be used by readers for generating abstracts [32]. Therefore, we assume that the intensity of cause-effect representation plays a positive role on identifying the important sentences within the section they belong to. Specifically, we obtain the following property, which is verified in S6 Appendix.

Property. *The intensity of cause-effect representation is in nonnegative correlation to the quality of the summary of the text they belong to*.

**Proposition 2. Cause-Effect Links within a Text Cover its Key Words**.

This can be verified by calculating the coverage of the *cause-effect* links within papers on the words of their abstracts and conclusions, which contain the key words of the papers from the author’s point of view.

Table 4 shows that the annotated *cause-effect* links cover 65% of the key words collected from *abstract* and *conclusion* on average, i.e., *cause-effect* links cover a majority of key words within scientific paper. This indicates that the *cause-effect* link connects the key concepts to render the core of text.

**Table 4:** The key words coverage of the annotated *cause-effect* links.

Article ID	Abstract (%)	Conclusion (%)	Abs&Conc (%)
f0001	84.17	69.84	76.83
f0002	79.03	54.12	64.63
f0003	85.03	87.23	86.39
f0014	0	0	0
f0015	0	74.29	74.29
f0016	58.21	0	58.21
f0027	38.89	0	38.89
f0028	0	74.04	74.04
f0029	50	43.82	46.99
Average	65.89	67.29	65.07

In Table 4, the zeros are generated from the absence of *abstract* or *conclusion* sections, and the coverage of paper f0027 is lower because the paper has no *abstract* and we treat subheadings as *abstract* to calculate coverage. The calculation uses exact matching without considering the synonyms of the extended key words. Using thesaurus will apparently improve the result.

## Automatic extraction of cause-effect links

To show the contribution of *cause-effect* link to representing the core of scientific paper, we implement a pattern-based algorithm for automatically extracting *cause-effect* links from more papers, and then use the *cause-effect* links to generate the summaries of the papers.

### Properties for extracting cause-effect link

From observation, we find two properties about the language expression of *cause-effect* link, which can be used to extract *cause-effect* links from text.

**Property**. *The cause component and the effect component of a cause-effect link are mainly positioned within the same sentence or between adjacent sentences*.

This can be observed from Table 5, which shows the percentage of the relative position of the cause component and the effect component within the annotated *cause-effect* links. The word *Adjacent* denotes a *cause-effect* link that the cause and the effect are in the same sentence or within adjacent sentences, and *Not-adj&Multi* denotes a *cause-effect* link that either consists of more than two sentences or the cause is not adjacent to the effect.

**Table 5:** The position of cause component and the effect component within the annotated *cause-effect* links.

Article ID	Adjacent (%)	Not-adj&Multi (%)
f0001	80.43	19.57
f0002	96.43	3.57
f0003	93.10	6.90
f0014	91.67	8.33
f0015	88.24	11.76
f0016	91.43	8.57
f0027	91.30	8.70
f0028	89.58	10.42
f0029	94.44	5.56
Average	90.74	9.26

On average, 90% of the annotated *cause-effect* links are on single sentences or within adjacent sentences. This reflects the fact that putting a cause and an effect consecutively enables authors to quickly represent a complete opinion and enables readers to understand quickly. This also verifies the efficiency principle and locality principle proposed in [6].

Property. Most cause-effect links have causal cues.

Causal cues refer to representative words or phrases such as “*because*” and “*due to*” that indicate *cause-effect* relations and connect a cause component to an effect component. Table 6 shows that 85% of the annotated *cause-effect* links have causal cues on average.

**Table 6:** The percentage of the annotated cause-*effect* links containing causal cues.

Article ID	Have causal cue (%)	No causal cue (%)
f0001	90.22	9.78
f0002	89.29	10.71
f0003	93.79	6.21
f0014	88.89	11.11
f0015	76.47	23.53
f0016	85.71	14.29
f0027	73.91	26.09
f0028	81.25	18.75
f0029	88.89	11.11
Average	85.38	14.62

### Extraction algorithm and result analysis

The above two properties support the design of a pattern-based algorithm (with reference to [51]) for automatically extracting *cause-effect* links whose cause component and effect component are within the same sentence or two adjacent sentences.

S3 Appendix gives the experimental details of the pattern-based *cause-effect* link extraction algorithm. S3 Appendix shows the design of syntactic patterns. S3 Appendix shows the procedures of using the syntactic patterns to extract *cause-effect* links from sentences. S3 Appendix illustrates some *cause-effect* links extracted from paper f0001. S3 Appendix classifies all the false-positive *cause-effect* links automatically extracted from the *OBSERVATION* dataset to further analyze the performance of the algorithm.

Table 7 shows that the automatic extraction algorithm gets 70% *Recall* on the *OBSERVATION* dataset on average. Besides, the syntactic patterns used in the algorithm are based on the causal cues observed from papers f0001, f0002 and f0003. However, we can see from Table 7 that the algorithm also performs well on the rest papers of the *OBSERVATION* dataset. This indicates that the causal cues we observed are commonly used by different authors, and that the language expressions of the *cause-effect* link used by different authors have no big difference.

**Table 7:** The performance of the *cause-effect* link extraction algorithm on the *OBSERVATION* dataset.

Article ID	Precision (%)	Recall (%)	F1-score
f0001	42.36	85.92	56.74
f0002	45.24	76	56.72
f0003	39.52	75.97	51.99
f0014	42.5	56.67	48.57
f0015	40	80	53.33
f0016	37.5	60	46.15
f0027	50	52.94	51.43
f0028	48	64.86	55.17
f0029	77.42	77.42	77.42
Average	46.95	69.98	55.28

The *Precision* of the algorithm is relatively lower in Table 7. We further invite annotators to classify all the false-positive *cause-effect* links extracted from the *OBSERVATION* dataset into 5 types, and then we find that 29.20% false-positive cases also properly express cause-effect relations but are missed by annotators. The precision of our algorithm increases to 59.7% and the F-score increases to 63.72% when correcting these wrong false-positive cases (see S3 Appendix in detail).

S4 Appendix shows the distribution of the extracted *cause-effect* links on the sections of each paper on the *EMY* dataset, and S5 Appendix shows the coverage of key words within the extracted *cause-effect* links on each paper of the *EMY* dataset. The results of S4 Appendix and S5 Appendixx verify that *proposition 1* and *proposition 2* hold on the larger dataset of journal papers. S7 Appendix and S7 Appendix further verify the two propositions on the *ACL2014* dataset and show that the two propositions also hold on the dataset of conference papers.

## The impact of cause-effect link on automatic summarization

To unveil the impact of the *cause-effect* link on summarization, we proposed four schemes for incorporating the annotated *cause-effect* links or the auto-extracted ones into nine benchmark summarization models to improve the quality of extractive summarization. Each of these benchmark models more or less uses some *is-part-of link* or *similar* link to build the instances of Semantic Link Network among language units (such as words, sentences, paragraphs and sections), uses these Semantic Link Network instances to determine the ranks of sentences, and extracts higher ranked sentences as automatically generated summary for each paper. ROUGE scores are used to evaluate the quality of the generated summaries [52]. The experiment on the *EMY* dataset shows the following effects of using *cause-effect link* (experimental details are given in S6 Appendix).

**Effect 1**. *Using the cause-effect link can improve the quality of the generated summaries*.

With any of the *cause-effect* link combination schemes, almost all ROUGE scores of the benchmark models are increased after incorporating *cause-effect* link into the building process of the Semantic Link Network instance or the sentence ranking process. The improvement is particularly prominent for structural benchmark models containing *is-part-of* link.

**Effect 2**. *Cause-effect link can identify important sentences*.

This is in line with a common sense: The important cause often leads to some important effects, vice versa. If a sentence is considered important and it is the cause or the effect of other sentences, it contributes more weight to the effect sentences or the cause sentences. Besides, we have known from proposition 2 that sentences on the *cause-effect* link cover most key words of a paper, so the higher ranked sentences involved *cause-effect* links may contain more important concepts than those sentences without *cause-effect* link. Thus, even using *cause-effect* links to filter the ranking result of the benchmark models can also get better performance.

**Effect 3**. *The core of papers can be better represented by semantic link network if more types of links are appropriately incorporated*.

The summaries generated by models incorporating more types of semantic links have higher quality. *This phenomenon verifies that Semantic Link Network with more types of semantic links can better represent the core of papers*. It also verifies the richness priority of emerging semantic links proposed in [2, 17].

**Effect 4**. *The intensity of cause-effect representation is in nonnegative correlation to the quality of the summary of the text they belong to*.

When the intensity of the cause-effect representation of a section reaches a certain level, summaries generated from this section by several sentence-ranking models achieve more satisfied quality than those sections with lower intensity of *cause-effect* link.

We further verify the above conclusions on the *ACL2014* dataset in S7 Appendix. The experimental results show that the above effects still hold on the dataset of conference papers.

We posted the source codes of the experiments of this paper in GitHub [53].

## Conclusion

This work verifies that the Semantic Link Network plays an important role in representing the core of scientific paper through the experiments on the contribution of the *cause-effect* link to representing the core of paper. The contribution of this work concerns the following aspects.

First, this work proposed and verified two propositions that reflect the importance of the *cause-effect* link: *The distribution of cause-effect links indicates the intensity of representation*, and the *cause-effect links cover the key words within text*. Verification carries out through observation, automatically extracting *cause-effect* links from scientific papers for supporting the propositions, and examining four schemes of incorporating the *cause-effect* link into the instances of Semantic Link Network for automatically generating the summaries for scientific papers. The experiments show that the quality of automatically generated summaries are improved after incorporating the *cause-effect* link, and the intensity of cause-effect representation has a significant effect on the quality of automatically generated summaries.

Second, this work deepens the understanding of how *cause-effect* link contributes to the core of text. This provides an evidence for developing advanced content services based on semantic link network such as automatic summarization, recommendation and question answering (e.g., finding the answer to the question through discovering and reasoning on semantic links such as *cause-effect* link, *is-part-of* link and *similarity* link) and inspires relevant research on Content Computing, Linked Data, Knowledge Graph, and Cyber-Physical Society [54].

## Supporting information

S1 Appendix The extensions of our previous work. (PDF)

S2 Appendix Principles for judging cause-effect links within text. (PDF)

S3 Appendix Experimental details of the Cause-effect Link extraction algorithm. (PDF)

S4 Appendix Distributions of cause-effect links on sections. (PDF)

S5 Appendix The coverage of key works on automatically extracted cause-effect links. (PDF)

S6 Appendix Experiments of automatic summarization with cause-effect link. (PDF)

S7 Appendix Extended experiments on ACL2014 dataset. (PDF)

S8 Appendix Details of the EMY dataset. (PDF)
