# Supplementary material for: The contribution of cause-effect link to representing the core of scientific paper—The role of Semantic Link Network
Source: PLoS One. 2018 Jun 21;13(6):e0199303. doi: 10.1371/journal.pone.0199303 (PMC6013162; doi:10.1371/journal.pone.0199303)
Supplement: S1 Appendix — (PDF) [file pone.0199303.s001.pdf]

# Appendix 1. The extensions of our previous work

This paper extends our previous work[1] on the observation, the experimental datasets, and the verifications of the role of semantic link in representing the core of text by applying the *cause-effect* link to improve automatic text summarization as shown in Table 8.

Table 8. The extensions of our previous work.

| | **Previous Work** | **Extensions** |
|---|---|---|
| **Experimental dataset** | *MY* dataset: It contains 13 journal papers (3 papers are annotated with cause-effect links). | **1.** *EMY* dataset: It contains 39 scientific papers, including 33 journal papers, 4 conference papers and 2 books (9 journal papers are annotated with cause-effect links and summaries of section).<br>**2.** *ACL2014* dataset: It contains 173 conference papers (88 long papers and 85 short papers) collected from the proceedings of ACL 2014. |
| **Observing the distribution and key word coverage of cause-effect links** | Observations were carried out on the annotated cause-effect links. Two propositions were proposed. | **1.** Six more papers with manually annotated cause-effect links were used for verifying observation.<br>**2.** Two propositions hold on the *EMY* dataset and the *ACL2014* dataset. |
| **Designing the algorithm to automatically extract cause-effect links** | Two properties of the language expressions of cause-effect link were observed from the annotated cause-effect links and used for designing the pattern-based algorithm to extract cause-effect links from the papers of *MY* dataset to verify the two propositions. | **1.** Two properties of the language expressions of cause-effect link still hold on 6 annotated papers.<br>**2.** The algorithm is applied to extract cause-effect links from the papers of the *EMY* dataset and the *ACL2014* dataset to verify the two propositions.<br>**3.** We further analysed the false-positive cases of the extracted cause-effect links to learn more about the performance of the automatic extraction algorithm. |
| **Verifying the role of Cause-effect link in rendering the core of papers by Automatic Summarization** | (Our previous work did not address this issue) | All the annotated and extracted cause-effect links were used in the automatic text summarization experiments to demonstrate the contribution of the cause-effect links to rendering the core of scientific papers. |

[1] Cao M, Sun X, Zhuge H. The Role of Cause-Effect Link Within Scientific Paper. Proceedings of the 12th International Conference on Semantics, Knowledge and Grids (SKG); Beijing: IEEE; 2016. pp. 32-9.
