# Supplementary material for: The contribution of cause-effect link to representing the core of scientific paper—The role of Semantic Link Network
Source: PLoS One. 2018 Jun 21;13(6):e0199303. doi: 10.1371/journal.pone.0199303 (PMC6013162; doi:10.1371/journal.pone.0199303)
Supplement: S2 Appendix — (PDF) [file pone.0199303.s002.pdf]

Appendix 2. Principles for judging cause-effect links within text

Annotators manually judge whether a text snippet *A* is the cause of a text snippet *B* according to the following three principles. Examples of *cause-effect* links are from paper f0002:

1) Phenomenon (or action) *A* is a reason for phenomenon (or hypothesis, or assertion) *B* to be true. For example:

- "*It goes through human brains for knowing, invention, propagation, fusion, generalization, and problem solving" →"knowledge is dynamic"*
- *"Knowledge flow networking can generate knowledge during operation" → "using the rules of knowledge flow makes teamwork more effective and innovative, not only in an e-science environment but also in team management"*

2) Condition *A* is a sufficient condition for phenomenon (or hypothesis, or assertion) *B*. For example:

- *"Knowledge flows in a network" → "knowledge flow spirals are formed"*
- *"Every node can innovate and output new knowledge to appropriate members" → "an effective knowledge flow network enables a research team to be very powerful in generating new knowledge"*

3) Executing action (or strategy) *A* will lead to the happening of phenomenon (or action, or "enabling" assertion) *B*. For example:

- *“Scientists can publish in several areas, and thus be involved in different knowledge flow network” → “knowledge to flow through knowledge networks in different areas to promote interdisciplinary research”*
- *“Encouraging unselfish cooperation”→ “help a knowledge flow network reach its greatest effectiveness”*
