# Supplementary material for: The contribution of cause-effect link to representing the core of scientific paper—The role of Semantic Link Network
Source: PLoS One. 2018 Jun 21;13(6):e0199303. doi: 10.1371/journal.pone.0199303 (PMC6013162; doi:10.1371/journal.pone.0199303)
Supplement: S3 Appendix — (PDF) [file pone.0199303.s003.pdf]

Appendix 3. Experimental details of the cause-effect link extraction algorithm

A. The design of syntactic patterns

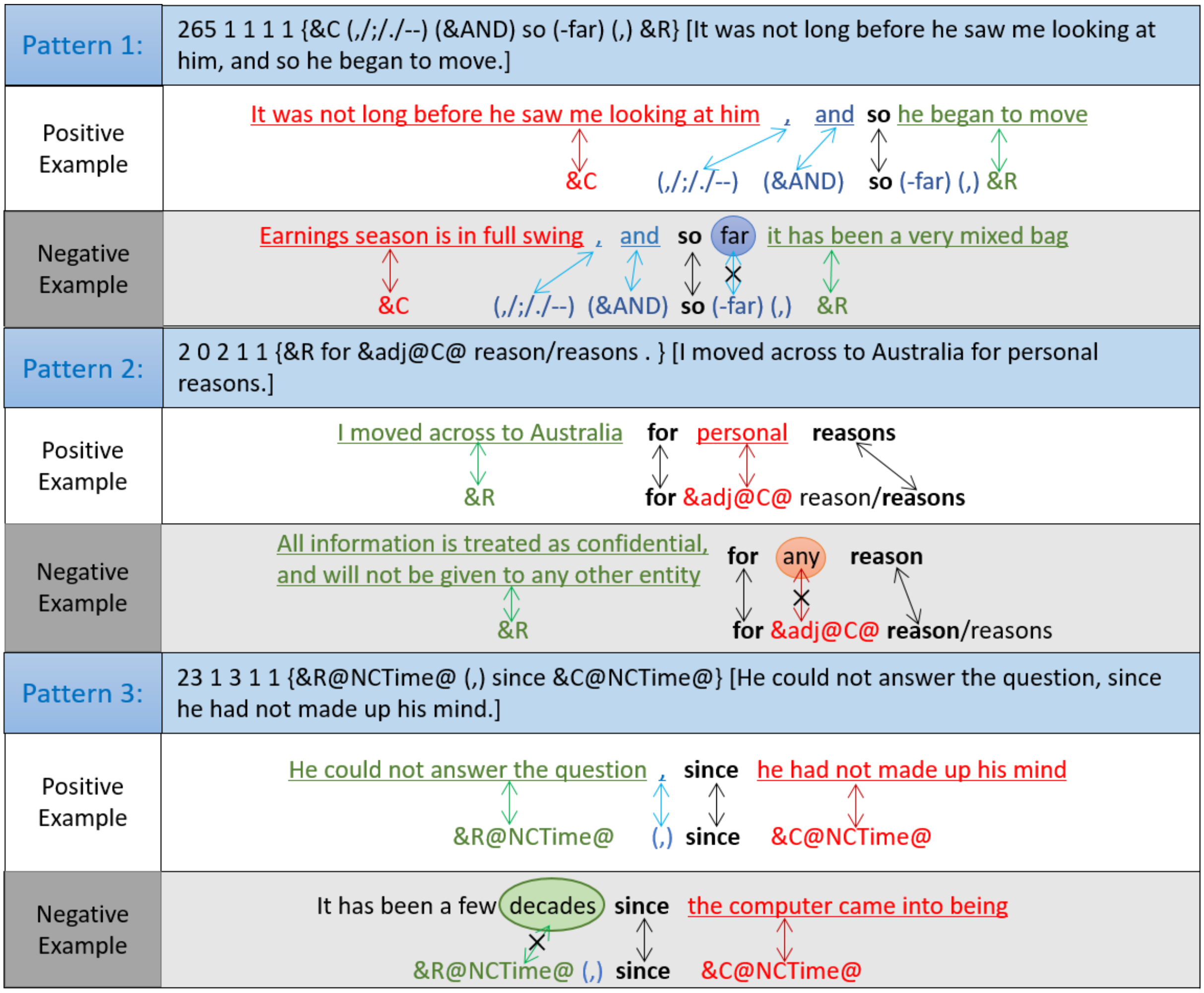

Fig 2. The design of cause-effect syntactic patterns.

We designed 145 syntactic patterns. Fig 2 shows three exemplars. Each pattern contains the following two main parts:

1) Five numbers that show the frequency, sentence processing capacity, and the category labels of the pattern.

The first number is the frequency of the causal cues. The second number indicates whether the pattern can extract a *cause-effect* link from two adjacent sentences. The third number to the fifth number represent the category labels of the pattern according to the taxonomy proposed in [55]. (The supplemental references of Appendix 3 are listed at the end of the Appendix.)

2) Main tokens and constraints that form the main body of the pattern.

The main body of each pattern contains two kinds of tokens: *main tokens* (i.e., words or phrases that can serve as a causal cue) that must be exactly matched, and *constraint tokens* that are used for restricting the context around the *main tokens* to help determine a *cause-effect* link by the part-of-speech of words, the syntax structure of the sentence, and the sense of noun-phrases.

In Fig 2, tokens within *"{}"* compose the main body of a pattern. The tokens starting with decorator *'&', '@'* and *'-'* are *constraints*, and those words without any decorators are *main tokens*. Two additional symbols are designed to extend the matching capabilities of the pattern: (1) the symbol '/' lists all alternative tokens for matching, and (2) the symbol '*()*' is bracket tokens that can be omitted in matching process. Each pattern definition can also have an exemplar sentence tagged by *"[ ]"* for examining the correctness of the token definition.

Table 9 shows all the causal cues used as the *main tokens* of syntactic patterns. Through observing the annotated *cause-effect* links of papers f0001, f0002 and f0003, we add 15 causal cues to those from [55], including causal words and their frequency through analyzing *cause-*

effect links in the London-Lund Corpus of Spoken English and the Lancaster-Oslo/Bergen Corpus of British English.

TABLE 9. The causal cues used as the *main tokens* of our syntactic patterns.

**Source**	**Causal Cues**		
From reference [55]	because	because of	therefore
	thus	hence	so
	so that	so &adj/&adv that	so as to
	as	since	due to
	accordingly	consequently	as a consequence
	in consequence	&ONE consequence of/to	owing to
	on that account	on account of	for the sake of
	have/has/had &NUM effect/effects	&V-ing , …	⋯ for &V-ing
	&ONE effect/effects of … is …	&ONE motivation/explanation/conclusion is (that)	
	with the result that	as/have/has/had &ONE/&NUM result/results (of)	
	by/for (&ONE) reason/reasons of	for &THIS/&ONE/&NUM reason/reasons	
	&ONE reason is that/because	it is for &THIS reason/reasons (,/that)	
	&ONE result is	&ONE/&NUM reason/reasons why/for	
	on … grounds	on (the/&THIS/&NUM) ground/grounds (,/./:/of/that)	
	&BE &ONE strategy	there &BE &NUM ground/grounds for (doing)	
	&THIS &BE why	(&ONE reason) why … because/&BE (that)	
	&THIS &BE not the only reason why	what conclusions can be drawn from … ?	
From the annotated cause-effect links of papers f0001, f0002 and f0003	if	if/once … ,/then …	based on
	according to	by/through &V-ing	lead/leads/led to
	indicate/indicates/indicated	realize/realizes/realized	ensure/ensures/ensured
	concern/concerns/concerned	imply/implies/implied	require/requires/required
	request/requests/requested	enable/enables/enabled	mean/means

Three types of *constraint tokens* are composed:

(1) *Syntax constraint* (the token starting with *'&'*), which defines the syntactic structure of the matched text snippet and further determines whether the matched text snippet serves as the cause component or the effect component. Taking *'&C'* of *Pattern 1* in Fig 2 for example, the matched text snippet should be a clause (i.e., containing either a subject-

predicate structure or a predicate–object structure) and it should be served as the cause component of a *cause-effect* link if matched. Stanford-parser is used for parsing part-of-speech tags and the syntax tree from sentence [56].

(2) *Subsidiary constraint* (the token starting and ending with *"@"*), which always follows a *Syntax constraint* to add additional restrictions on the matched text snippet. Taking '*&adj@*C*@*' of *Pattern 2* in Fig 2 for example, *"&adj"* requests that the part-of-speech tag of the matched word should be "*JJ**" (representing an adjective word) and the subsidiary constraint "*@C@*" further requires that the matched adjective word is the cause of a *cause-effect* link. Taking "*&R@NCTime@*" of *Pattern 3* for another example, "*&R*" matches a clause that serves as an effect, and "*@NCTime@*" further requests that the word sense of noun-phrases in the matched clause should not represent a time span. WordNet is used to get the word sense of nouns.

(3) *Preclusive constraint* (tokens starting with '-'), which prohibits the appearance of some words. Taking "*-far*" of *Pattern 1* for example, it requires that the word following the *main token* "*so*" should not be "*far*".

Table 10 lists all the *Syntax* constraints and the *Subsidiary* constraints used in our syntactic patterns.

TABLE 10. *Syntax* and *Subsidiary* constraints in our syntactic patterns.

**Type of Constraints**	**Constraint Tokens**	**The request for the matched text snippet**
Syntax constrains	&C	A clause that serves as the cause
	&R	A clause that serves as the effect
	&Clause	An attributive, appositive or declarative clause
	&ClauseHead	[that, whether, if, what, whatever, who, whoever, whom, whose, which, when, where, how, why]
	&THIS	[this, that, these, those, it, which]

	&this	[this, that, these, those]
	&ADJ	[direct, same, main, separate, final, alone, important, major, key, biggest, possible, only, primary]
	&adj	A word sequence whose POS tag started with "*JJ*"
	&ADV	[just, also, only, merely, all, alone, often, simply]
	&adv	A word sequence whose POS tag started with "*RB*"
	&NP	A noun-phrase which is labeled as "*NP*" by the Standford-parser
	&N	A word sequence whose POS tag started with "*NN*"
	&V-ing	A gerundial phrase which is labeled as "*V-ing*" + "*NP*" by the Standford-parser
	&V	A word whose POS tag started with "*V*"
	&TODO	An infinitive phrase which is labeled as "*TODO*" + "*NP*" by the Standford-parser
	&CAN	[can, could, may, might, will, would, must, should, ought to]
	&BE	[be, is, are, was, were,being, been, have been, 'v been]
	&AND	[and, but, so, also]
	&ONE	[the,a,an,one,another]
	&OF	[one of, part of]
	&NUM	[1,2,3,...,one,two,three,....,a, an, several,a few,some, many, part]
	&MODNUM	[at least, at most]
Subsidiary constrains	@C@	The text snippet is the cause
	@R@	The text snippet is the effect
	@NCTime@	There does not exit a noun-phrase in the text snippet represents a time span
	@NCPeople@	There does not exit a noun-phrase in the text snippet represents persons
	@Complete@	A clause must has predicate and subject (the object does not exist for an imperative sentence)

B. The extraction algorithm

The algorithm for automatically extracting the *cause-effect* links from scientific papers based on the predefined syntactic patterns is shown in Algorithm I.

In the procedure *GetPtList*, all the syntactic patterns are sorted to form a pattern list according to the generalization of the *main tokens* (a *main token* is more general than another

main token if it is a sub-string of the later token, e.g., *"because"* is more general than *"because of"*), the number of *constraints* and the frequency of a pattern.

In the procedure *GetCEList*, for each sentence of a paper, the algorithm sequentially matches each pattern in the pattern list against a sentence to find a suitable pattern for extracting a *cause-effect* link contained in this sentence.

Algorithm 1 Automatic Cause-effect Link Extraction Algorithm

Input: a paper dataset D, the text file of manually wittern patterns $manual\_pattern.txt$.
Output: the pattern list $PtList$, the cause-effect link list $CEList$.

```
function GetPtList(manual_pattern.txt)
    PtList ← [ ]
    for each text line ptxt in manual_pattern.txt do
        if ptxt is a manually designed pattern then
            (freq_type, main_tokens, constrains, examples) ← ParsePtTxt(ptxt)
            pt ← GetPatternObj(freq_type, main_tokens, constrains, examples)
            PtList.append(pt)
        end if
    end for
    PtList ← Sorted(PtList)
    return PtList
end function

function GetCEList(PtList, D)
    CEList ← [ ]
    for each paper in D do
        TempCEList ← [ ]
        SentTxtList ← GetSentenceTextList(paper)
        for sid in range(1, length(SentTxtList) do
            stxt ← SentTxtList.Get(sid)                    ▷ Get(sid)=NULL for IndexError
            stxt_l ← SentTxtList.Get(sid-1)
            stxt_r ← SentTxtList.Get(sid+1)
            for each pt in PtList do
                if FindMainTokens(pt.main_tokens, stxt) = False then
                    Continue
                end if
                (stxt_list, cause, effect) ← MatchConstrain(pt.constrains, stxt_l, stxt, stxt_r)
                if stxt_list is empty list then
                    Continue
                end if
                celink ← CreatCELinkObj(pt, stxt_list, cause, effect)
                TempCEList.append(celink)
                Break
            end for
        end for
        CEList.append(TempCEList)
    end for
    return CEList
end function
```

C. Auto-extracted cause-effect links

Table 11 shows a set of auto-extracted *cause-effect* links from paper f0001. The column "*Sent ID*" is the ID of sentence in paper f0001, "*pt*" is the cause-effect patterns written in *pattern frequency* + *main tokens* + *constraints* form, and "*stxt*" is the text of the sentences from which the *cause-effect* link is extracted.

In Table 11, case 50 and case 54 are true-positive (labeled by annotators), while case 51, case 52 and case 53 are false-positive. However, we find that case 51 in fact properly expresses a cause-effect relation according to the principles in Appendix 2 but it is missed by the annotators. The performance of the cause-effect link extraction algorithm is improved after correcting these wrong false-positive cases (as shown in Appendix 3.4).

TABLE 11. A set of auto-extracted cause-effect links from paper f0001.

Case ID	Sent ID	Pattern & Sentence & Cause & Effect	
50	292	**pt**	45 [['so', 'that']] ---- [['&C'], ['&R']]
		**stxt**	In scientific papers, the core representations usually appear in the front and in the end -LRB- e.g., title, abstract and conclusion -RRB- so that readers can be impressed before and after reading the main body.
		**cause**	In scientific papers, the core representations usually appear in the front and in the end -LRB- e.g., title, abstract and conclusion -RRB-
		**effect**	readers can be impressed before and after reading the main body.
51	293	**pt**	0 [[['by', 'through']]] ---- [['&R@Complete@'], ['&V-ing@C@']]
		**stxt**	This helps enhance the memory of the core by focusing and refocusing on the core when building or retrieving the semantic images in the mental space.
		**cause**	focusing and refocusing on the core when building or retrieving the semantic images in the mental space
		**effect**	This helps enhance the memory of the core
52	294	**pt**	265 [['so']] ---- [['&C', '(,/;/./--)', '(&AND)'], ['(-far)', '(,)', '&R']]
		**stxt**	Humans have been composing complex representations and making summarization through times, so we have the following axiom.
		**cause**	Humans have been composing complex representations and making summarization through times
		**effect**	we have the following axiom.
53	297<br>298	**pt**	62 [['therefore']]---- [['&C', '(,/;/./--)', '(&AND)'], ['(,)', '&R']]
		**stxt**	This axiom is the basis of representation -LRB- including using languages -RRB- and summarization -LRB- especially , for multi-document summarization -RRB- . Therefore , a representation p can be formalized as a structure of representations :
		**cause**	This axiom is the basis of representation -LRB- including using languages -RRB- and summarization -LRB- especially , for multi-document summarization -RRB-
		**effect**	a representation p can be formalized as a structure of representations :

**54**	**306**	**pt**	265 [['so']]---- [['&C', '(,/;/./--)', '(&AND)'], ['(-far)', '(,)', '&R']]
		**stxt**	Dictionaries explain words in texts , so they can be regarded as the basic implicit citations to all texts .
		**cause**	Dictionaries explain words in texts
		**effect**	they can be regarded as the basic implicit citations to all texts .

D. Analysis of false-positive cause-effect links

We analyzed each false-positive *cause-effect* link automatically extracted from the *OBSERVATION* dataset, and classified the causes of the false-positive cases into five types:

1) *Matching-Order*. The algorithm only uses the first matched pattern of the pattern list for extracting a *cause-effect* link from a sentence even when the pattern is not the most suitable pattern for the sentence.

2) *Sentence-Number*. Based on the property that *cause and effect components of a cause-effect link are mainly positioned within the same sentence or between two adjacent sentences*, the algorithm uses at most two sentences for extracting a *cause-effect* link. Therefore, the *cause-effect* links expressed by more than two sentences cannot be properly extracted.

3) *Pattern-Limitation*. The text expression of a sentence is flexible while our predefined syntactic patterns are fixed. So the syntactic patterns may not be able to correctly extract a cause or an effect. Moreover, whether a sentence matches the *constrains* of a pattern depends on the syntactic tree of the sentence parsed by Stanford-parser, and the incorrect parsing will lead to a false-positive *cause-effect* link.

4) *Wrong-False-Positive*. Some false-positive *cause-effect* links properly express cause-effect relations. They are missed by the annotators but are correctly extracted by the algorithm.

5) *No-Cause-Effect*. These false-positive *cause-effect* links do not express the cause-effect relations.

Table 12 shows the percentage of each type of false-positive *cause-effect* links automatically extracted from the *OBSERVATION* dataset. The percentage of the *cause-effect* links missed by the annotators is 29.20%. Table 13 shows the updated *precision, recall,* and *F-score* when we add these previously missed cause-effect links to the manually annotated cause-effect links. The *precision* score is raised by 27.16% compared to the previous test and the overall *F-score* is improved by 15.26%.

TABLE 12. The percentage of five types of the false-positive *cause-effect* links.

Article ID	Matching-Order	Sentence-Number	Pattern-Limitation	Wrong-False-Positive	No-Cause-Effect
f0001	6.0241	10.8434	6.0241	26.506	50.6024
f0002	8.6957	0	4.3478	65.2174	21.7391
f0003	12.1795	3.2051	5.7692	32.0513	46.7949
f0014	34.7826	8.6957	13.0435	26.087	17.3913
f0015	16.6667	10	20	16.6667	36.6667
f0016	13.3333	6.6667	10	20	50
f0027	44.4444	0	0	22.2222	33.3333
f0028	19.2308	7.6923	7.6923	26.9231	38.4615
f0029	14.2857	0	14.2857	0	71.4286
Average	13.6951	5.9432	7.7519	**29.199**	43.4109

TABLE 13. The performance of the extraction algorithm after correcting the wrong false-positive cases.

Article ID	New Precision (%)	New Recall (%)	New F1-score
f0001	56.46258503	72.80701754	63.60153257
f0002	80.95238095	79.06976744	80
f0003	59.67741935	75.8974359	66.81715576
f0014	57.5	54.76190476	56.09756098
f0015	50	64.1025641	56.17977528
f0016	50	58.53658537	53.93258427
f0027	61.11111111	44	51.1627907

f0028	60.78431373	56.36363636	58.49056604
f0029	77.41935484	66.66666667	71.64179104
Average	**59.7037037**	68.30508475	**63.71541502**

Supplemental references

55. Altenberg B. Causal Linking in Spoken and Written English. Studia linguistica. 1984; 38(1):20-69.
56. Klein D, Manning CD. Accurate Unlexicalized Parsing. Proceedings in the 41st Meeting of the Association for Computational Linguistics (ACL) 2003. pp. 423-30.
