# Supplementary material for: The contribution of cause-effect link to representing the core of scientific paper—The role of Semantic Link Network
Source: PLoS One. 2018 Jun 21;13(6):e0199303. doi: 10.1371/journal.pone.0199303 (PMC6013162; doi:10.1371/journal.pone.0199303)
Supplement: S4 Appendix — (PDF) [file pone.0199303.s004.pdf]

Appendix 4. Distributions of cause-effect links on sections

Table 14 lists the distribution of the manually annotated *cause-effect* links and auto-extracted *cause-effect* links on each section of a paper in the *OBSERVATION* dataset. Column *Article ID* is the ID of a paper, *Sent Num* lists the number of sentences in each section, *Annotated Cover Rate* is the percentage of sentences which contain manually annotated *cause-effect* links, and *Extracted Cover Rate* is the percentage of sentences which contain auto-extracted *cause-effect* links.

Distribution information in Table 14 shows that the sections with a higher *Annotated Cover Rate* also have a relatively higher *Extracted Cover Rate*. So the auto-extracted *cause-effect* links can be used to verify Proposition 1.

Table 15 shows the distribution of auto-extracted *cause-effect* links on each section of the rest 30 papers in the *EMY* dataset. It shows that *the sections with a higher intensity of the cause-effect representation also have a higher Extracted Cover Rate*. Thus, Proposition 1 still holds on the larger dataset of journal papers.

Table 14. The distribution of the annotated and extracted *cause-effect* links on the *OBSERVATION* dataset.

Article ID	Section ID	Section Title	Sent Num	Annotated Cover Rate	Extracted Cover Rate
f0001	0	Abstract	12	25.0000	0.0000
	1	Introduction	118	9.3220	17.7966
	2	Multi-Dimensional Methodology	36	5.5556	11.1111
	3	Basic Characteristics and Principles of Language Use …	70	98.5714	34.2857
	4	General Citation - Definition, Measure and Axiom	81	28.3951	29.6296
	5	Dimension of Representation	99	14.1414	19.1919
	6	Multi-Dimensional Evaluation	38	23.6842	21.0526
	7	Incorporating pictures into summary	77	24.6753	31.1688
	8	Summarizing Videos, Graphs and Pictures	75	26.6667	26.6667
	9	General Summarization	93	17.2043	23.6559
	10	Conclusion	13	7.6923	15.3846
f0002	0	Abstract	5	0.0000	20.0000
	1	Introduction	19	5.2632	0.0000
	2	Knowledge Flows through a Citation Network	11	36.3636	45.4545
	3	The Knowledge Flow Spiral—A Knowledge …	54	14.8148	38.8889

	4	Using and Managing Knowledge Flow Networks in …	48	29.1667	29.1667
	5	Knowledge Flows through Semantic Link Networks	24	8.3333	25.0000
	6	Conclusion	6	16.6667	16.6667
f0003	0	Abstract	12	41.6667	25.0000
	1	Introduction	214	17.2897	23.8318
	2	Cyber-Physical-Physiological-Psychological-Socio- …	110	25.4545	25.4545
	3	The evolving complex space	66	15.1515	28.7879
	4	Interaction pattern and behavior principle in complex …	44	13.6364	20.4545
	5	Symmetry, self-similarity and multi-level semantic …	37	21.6216	13.5135
	6	The mental space	66	10.6061	24.2424
	7	The Semantic Link Network	141	17.7305	22.6950
	8	Linking, interacting and explaining through spaces	112	3.5714	13.3929
	9	Complex link network and socio energy	91	15.3846	21.9780
	10	Closed loops of complex link	49	26.5306	28.5714
	11	The interactive co-computing environment	57	10.5263	24.5614
	12	Philosophical perspective	88	15.9091	25.0000
	13	Summary	19	36.8421	21.0526
f0014	1	Introduction	17	52.9412	70.5882
	2	What is meant by efficiency	25	68.0000	60.0000
	3	How to design machines making use …	36	27.7778	19.4444
	4	Remarks	24	33.3333	29.1667
f0015	1	Introduction	19	10.5263	42.1053
	2	The principle of rationality	18	11.1111	16.6667
	3	Mental qualities of machines	32	34.3750	31.2500
	4	Approximate criteria of some mental qualities	30	26.6667	16.6667
	5	Examples	56	28.5714	30.3571
	6	Attitudes aimed at understanding a system	28	10.7143	14.2857
	7	Conclusion	11	36.3636	18.1818
f0016	0	Abstract	56	28.5714	26.7857
	1	WHAT IS COMMON SENSE?	4	0.0000	25.0000
	2	COMMON SENSE KNOWLEDGE	57	19.2982	31.5789
	3	COMMON SENSE REASONING	45	31.1111	37.7778
f0027	0	Abstract	2	0.0000	0.0000
	1	Introduction	22	22.7273	13.6364
	2	The same language	28	53.5714	39.2857
	3	Data gold-mine	30	40.0000	33.3333
	4	Smaller is faster	14	57.1429	14.2857
f0028	1	Data-intensive science -- a new paradigm	19	31.5789	26.3158
	2	New data-analysis methods	9	66.6667	33.3333
	3	Science centers	31	45.1613	19.3548
	4	Metadata enables data access	17	17.6471	29.4118
	5	Semantic convergence: numbers to objects	25	8.0000	8.0000
	6	Metadata enables data independence	33	30.3030	33.3333
	7	Set-oriented data access gives parallelism	34	14.7059	20.5882
	8	Other useful database features	11	27.2727	9.0909
	9	Ending the impedance mismatch	17	35.2941	11.7647
	10	What's wrong with files?	15	33.3333	26.6667
	11	Why scientists don't use databases today	21	66.6667	23.8095
	12	Why things are different now	7	0.0000	0.0000
	13	Some hints of success	20	5.0000	15.0000
	14	Summary	20	20.0000	20.0000
f0029	0	Abstract	12	0.0000	8.3333
	1	Terminology: Hits, Misses, Ephemeral, Masked, Edge	49	44.8980	22.4490
	2	Computing Match Hits	9	22.2222	22.2222
	3	Computing Match Misses	58	5.1724	8.6207
	4	Friends-of-Friends -- Match Transitive Closure	18	11.1111	0.0000

	5	Bundles	39	15.3846	10.2564
	6	SDSS Experience, Moving Objects, and Multi-Survey…	46	21.7391	17.3913
	7	Summary	10	0.0000	0.0000

Table 15. The distribution of extracted *cause-effect* links on the rest papers of the *EMY* dataset.

Article ID	Section ID	Section Title	Sent Num	Extracted Cover Rate
f0004	1	Introduction	24	12.50
	2	Papers in this Issue	20	25.00
	3	Summary	4	25.00
f0005	0	Abstract	12	8.33
	1	Introduction	136	16.91
	2	Semantic Link Network -- Weaving Implicit Web on …	133	11.28
	3	Multi-Dimensional Space on Data	96	25.00
	4	Multi-Dimensional Analytics	69	18.84
	5	Unconventional Mapping from Data into Knowledge …	263	22.43
	6	Cognitive Cyber-Infrastructure	104	9.62
	7	Communities of Cognition and Practice	60	13.33
	8	New Paradigm of Science	59	18.64
	9	The Nature of Big Data Computing	127	22.05
	10	Summary	19	21.05
f0006	0	Abstract	9	22.22
	1	Introduction	27	25.93
	2	Separation	38	18.42
	3	Semantic image and dimension	28	0.00
	4	Super link	19	15.79
	5	Influence	17	5.88
	6	Distinguished characteristics	38	5.26
	7	Scientific issues	50	24.00
	8	Comparison with Internet of Things and Cyber-Physic…	44	18.18
	9	Conclusion	54	24.07
f0007	1	Introduction	28	17.86
	2	Scientific issues	20	15.00
	3	Organization of this issue	26	26.92
	4	Summary	5	40.00
f0008	0	Abstract	9	22.22
	1	Introduction	58	20.69
	2	The Resource Space Model RSM	94	22.34
	3	The Probabilistic Resource Space Model P-RSM	155	11.61
	4	Operations Of Probabilistic Resource Space Model	75	17.33
	5	Probabilistic Integrity Constraints	57	8.77
	6	Analysis	64	31.25
	7	Extensions	107	24.30
	8	Application Examples	69	13.04
	9	Conclusion	12	33.33
f0009	0	Abstract	5	40.00
	1	Introduction	68	22.06
	2	Characteristics of Minimum Semantic Cover and …	32	37.50
	3	Basic Operations and Characteristics of Cpsocio-SLN	112	30.36
	4	Maintenance and Query	136	37.50
	5	An Example on Basic Operations	15	6.67
	6	Summary	12	50.00
f0010	0	Abstract	8	12.50

	1	Introduction	52	34.62
	2	Document SLN	80	22.50
	3	Content-Based Semantic Link Discovery	50	22.00
	4	Discovering Semantic Links According To Attribute …	17	23.53
	5	Reasoning	17	29.41
	6	Evolution	71	22.53
	7	Conclusion	9	11.11
f0011	0	Abstract	18	33.33
	1	Introduction	91	17.58
	2	Semantic Worldview	79	13.92
	3	Building and Explaining Semantic Image	96	18.75
	4	Interactive Semantic Base	51	19.61
	5	Interactive Semantics = Interactive System + Semantic …	62	24.19
	6	Semantic Lens	106	21.70
	7	Conclusion	29	17.24
f0012	1	Introduction	38	7.89
f0013	0	Abstract	7	14.29
	1	Introduction	40	17.50
	2	Schema for The Semantic Link Network	36	0.00
	3	SLN Operations	86	4.65
	4	Rule-Constraint Normal Forms for The SLN Schema	195	9.74
	5	Schema Maintenance and Reasoning	96	14.58
	6	Case Study	95	16.84
	7	Discussion	12	8.33
	8	Conclusions	5	20.00
f0017	1	Introduction	67	34.3284
	2	The Construction of the Advice Taker	121	12.3967
f0018	1	INTRODUCTION	45	46.6667
	2	EPISTEMOLOGICAL PROBLEMS	77	31.1688
	3	CIRCUMSCRIPTION -- A WAY OF JUMPING TO CONCLUSIONS	53	26.4151
	4	CONCEPTS AS OBJECTS	93	31.1828
	5	PHILOSOPHICAL NOTES	39	51.2821
f0019	0	Abstract	4	50.0000
	1	Introduction	34	38.2353
	2	The Philosophy of Artificial Intelligence	5	0.0000
	3	Epistemological Adequacy	5	20.0000
	4	Free Will	2	0.0000
	5	Natural Kinds	6	16.6667
	6	Four Stances	8	0.0000
	7	Ontology and Reification	6	0.0000
	8	Counterfactuals	5	40.0000
	9	Philosophical Pitfalls	4	0.0000
	10	Philosophers! Help!	20	20.0000
f0020	0	Abstract	11	45.4545
	1	Introduction	75	17.3333
	2	What kinds of approximate concepts are there?	180	14.4444
	3	Propositional approximate theories	32	12.5000
	4	When an approximate concept becomes precise	16	12.5000
	5	Conclusions, remarks, and acknowledgements	6	0.0000
f0021	0	Abstract	5	20.0000
	1	Algorithm = logic + control	12	33.3333
	2	The pereira{porto logic program	23	13.0435
	3	Reducing the map	17	58.8235
	4	Kempe transformations	18	38.8889
	5	Realizing the reduction algorithm by control of …	25	52.0000
	6	Realizing the kempe transformation algorithm	13	30.7692
	7	Acknowledgements	2	0.0000

f0022	0	Abstract	9	22.2222
	1	Introduction	8	50.0000
	2	The mutilated checkerboard	57	28.0702
	3	Pinning down the ideas, English first	40	30.0000
	4	Elementary first order formulations	55	12.7273
	5	Expressing the creative ideas in set theory	48	8.3333
	6	Acknowledgements	2	0.0000
f0023	0	Abstract	19	15.7895
	1	The Informal theory	27	29.6296
	2	Situation calculus formulas for SDFW	53	15.0943
	3	A generalization of SDFW	10	40.0000
	4	Knowledge of one's free will and wanting more or fewer ...	17	29.4118
	5	Philosophical issues	13	38.4615
	6	Praise and blame	16	31.2500
	7	A possible experiment with apes	28	14.2857
	8	Comparison with Dennett's ideas	20	20.0000
	9	Summary and remarks	45	22.2222
f0024	0	Abstract	18	16.6667
	1	REPRESENTING BEHAVIOR BY PROGRAM	12	33.3333
	2	THE GPS AND ITS SUCCESSORS	9	44.4444
	3	PRODUCTION SYSTEMS	21	38.0952
	4	REPRESENTING KNOWLEDGE IN LOGIC	68	23.5294
	5	NONMONOTONICITY	33	15.1515
	6	REIFICATION	8	12.5000
	7	FORMALIZING THE NOTION OF CONTEXT	30	26.6667
f0025	0	Abstract	8	25.0000
	1	Introduction	3	0.0000
	2	Features of the Formalism	34	23.5294
	3	Applications	24	12.5000
	4	Desiderata for a Mathematical Logic of Context	20	20.0000
	5	Remarks	11	9.0909
f0026	0	Abstract	6	0.0000
	1	Introduction	12	25.0000
	2	Basic Consciousness	11	0.0000
	3	Consciousness of Self	29	31.0345
	4	Moody Zombies	4	0.0000
f0030	0	Abstract	14	21.4286
	1	The Virtual Observatory	108	30.5556
	2	Web Services: Using Distributed Data	46	19.5652
	3	Hierarchical Architecture	41	4.8780
	4	The Virtual Observatory and the Grid	63	26.9841
	5	Outreach Using the Virtual Observatory	8	0.0000
	6	Summary	13	15.3846
f0031	0	Abstract	4	0.0000
	1	Introduction	74	21.6216
	2	The Time Dimension	27	22.2222
	3	Uniform Views of Diverse Data	32	34.3750
	4	Better Algorithms	13	7.6923
	5	Education	27	11.1111
f0032	1	Contents	41	7.3171
	2	Acknowledgments	1	0.0000
f0033	0	Abstract	3	0.0000
	1	A Thousand-Transactions-per-second was once difficult ...	16	12.5000
	2	DebitCredit on a PC?	34	20.5882
	3	Caveat: Why these tps results are bogus	22	40.9091
	4	Summary and Observations	23	34.7826

f0034	0	Abstract	3	33.3333
	1	Introduction	43	13.9535
	2	Science is Changing	49	20.4082
	3	Rules of Engagement -- Working With X	81	27.1605
	4	Architecture: Services and Portals.	47	25.5319
	5	Was It Worth the Effort?	36	30.5556
	6	Some Lessons Learned From the WWT	80	28.7500
	7	Summary	10	20.0000
	8	Acknowledgments	5	0.0000
f0035	0	Abstract	7	14.2857
	1	Introduction	27	7.4074
	2	Astronomy as an Archetype for Online Science	36	25.0000
	3	Astronomy Data Will All be Online	13	0.0000
	4	The World-Wide Telescope	36	22.2222
	5	The Virtual Observatory and SkyServer	48	16.6667
	6	Summary	10	20.0000
	7	Acknowledgments	4	0.0000
f0036	0	Abstract	5	0.0000
	1	Introduction	5	20.0000
	2	Storage performance and price	112	22.3214
	3	Amdahl's system balance rules	60	15.0000
	4	Networking: Gilder's Law	38	23.6842
	5	Caching: Location, Location, and Location	133	28.5714
	6	Summary	12	8.3333
	7	Acknowledgments	1	0.0000
f0037	1	Introduction	18	5.5556
	2	Functionality	7	42.8571
	3	Availability	68	30.8824
	4	Agility	21	14.2857
	5	Manageability	26	7.6923
	6	Scalability	76	25.0000
	7	Summary	14	28.5714
	8	Acknowledgments	1	0.0000
f0038	1	The Problem: How do we exchange Terabyte datasets?	14	50.0000
	2	Sneaker Net	9	22.2222
	3	The case against tape	11	18.1818
	4	Disk Bricks: Portable Terabytes	32	25.0000
	5	Storage Brick Cost:	7	14.2857
	6	Taxes and Shipping	3	66.6667
	7	Storage Brick Performance:	22	9.0909
	8	Storage Brick Security:	21	33.3333
	9	Compression and Encryption	8	75.0000
	10	Summary	5	20.0000
	11	Acknowledgments	1	0.0000
f0039	1	Introduction	28	35.7143
	2	How much information is there, and how much astronomy …	31	22.5806
	3	Moore's, Gilder's, Metcalf's, and Amdahl's Laws	11	36.3636
	4	Processors	66	36.3636
	5	Memory and the Memory Hierarchy	131	32.0611
	6	Communications	50	22.0000
	7	Software	82	21.9512
	8	Summary	9	44.4444
