# Supplementary material for: The contribution of cause-effect link to representing the core of scientific paper—The role of Semantic Link Network
Source: PLoS One. 2018 Jun 21;13(6):e0199303. doi: 10.1371/journal.pone.0199303 (PMC6013162; doi:10.1371/journal.pone.0199303)
Supplement: S5 Appendix — (PDF) [file pone.0199303.s005.pdf]

Appendix 5. The coverage of key words on automatically extracted cause-effect links

The *abstract* and *conclusion* are treated as standard texts for comparison. The coverage of key words on the *cause-effect* links is the percentage of the words that are not only listed on the *cause-effect* links but also within the compared standard texts to the compared standard texts.

Table 16 shows the coverage of the key words on the *cause-effect* links automatically extracted from the papers of the *EMY* dataset (the coverage of the key words within papers of the *OBSERVATION* dataset is shown in boldface letter). A "0" exception is listed due to the absence of *abstract* or *conclusion* section in the corresponding paper.

Auto-extracted *cause-effect* links cover 65% of the key words collected from *abstract* and *conclusion* on average. This verifies Proposition 2 that *cause-effect links within a text cover its key words* still holds on the larger dataset of journal papers.

TABLE 16. The coverage of key words on auto-extracted *cause-effect* links.

Article ID	Abstract (%)	Conclusion (%)	Abs&Conc (%)
**f0001**	**71.6667**	**73.0159**	**72.3577**
**f0002**	**88.7097**	**54.1176**	**68.7075**
**f0003**	**86.3946**	**88.5106**	**87.6963**
f0004	0	41.0256	41.0256
f0005	84.6154	84.2105	84.3066
f0006	75.6757	63.2319	65.0699
f0007	0	67.5676	67.5676
f0008	93.1034	83.3333	87.1111
f0009	75.8621	91.8699	86.7403
f0010	68.3333	78.0822	73.6842
f0011	88.5135	84.3972	85.814
f0012	0	0	0
f0013	61.2245	82.5	70.7865
**f0014**	**0**	**0**	**0**
**f0015**	**0**	**65.7143**	**65.7143**
**f0016**	**62.8019**	**0**	**62.8019**
f0017	0	0	0
f0018	0	0	0
f0019	77.2727	0	77.2727
f0020	75.5556	17.7778	56.2963

f0021	65.3846	0	65.3846
f0022	77.7778	0	77.7778
f0023	63.4783	54.4218	56.9682
f0024	55.7971	0	55.7971
f0025	44.2857	0	44.2857
f0026	32.7869	0	32.7869
**f0027**	**27.7778**	**0**	**27.7778**
**f0028**	**0**	**70.1923**	**70.1923**
**f0029**	**53.1915**	**49.4624**	**51.3369**
f0030	58.209	65.3226	61.6279
f0031	56.4103	0	56.4103
f0032	0	0	0
f0033	31.8182	60.9302	58.2278
f0034	74.2857	0	74.2857
f0035	68	0	68
f0036	68.6275	0	68.6275
f0037	0	0	0
f0038	0	0	0
f0039	0	87.2727	87.2727
Average	66.20591	68.14782	65.92849
