# Supplementary material for: The contribution of cause-effect link to representing the core of scientific paper—The role of Semantic Link Network
Source: PLoS One. 2018 Jun 21;13(6):e0199303. doi: 10.1371/journal.pone.0199303 (PMC6013162; doi:10.1371/journal.pone.0199303)
Supplement: S6 Appendix — (PDF) [file pone.0199303.s006.pdf]

Appendix 6. Experiments of automatic summarization with cause-effect link

A. Benchmark summaries for model evaluation

We use *cause-effect* links to produce summaries for each paper. By evaluating the quality of summaries, we show that *cause-effect* link is important and effective in representing the core of paper. When summarization is conducted on a paper, its *abstract*, *conclusion*, and their combination are regarded as three benchmark summaries. Sentences belonging to *abstract* or *conclusion* are removed from the input of the summarization models. Qualities of automatic summaries generated by different models are evaluated by comparing the ROUGE scores.

Annotated *cause-effect* links are used to produce summary on the *OBSERVATION* dataset. Auto-extracted *cause-effect* links are used on the *EMY* dataset and the *ACL2014* dataset, and the details on the *ACL2014* dataset are given in Appendix 7.3.

An automatically generated summary should not be longer than the average length of three benchmark summaries and should contain as many sentences as possible. Taking paper f0001 for example, its *abstract* section contains 221 words, and its *conclusion* section contains 231 words. The length limitation of a produced summary of paper f0001 is $\frac{221+231+(221+231)}{3}$ =301 words.

B. Summarization models for comparisons

Nine benchmark models for single paper summarization are compared. They use *is-part-of* or *similarity* links to build the instances of Semantic Link Network among textual expression units (words, sentences, paragraphs and sections) for sentence ranking. Top-k ranked sentences are automatically extracted as the summary. The following are these benchmark models.

1) Model *TF-IDF* uses the sum of TF-IDF weights of words within a sentence as the weight of the sentence.

2) Model *GS* views sentences as nodes and connects sentences with *similar* links weighted by *Jaccard* distance between two sentence strings to form a *similarity graph*. A Page-Rank algorithm is applied to the *graph* of sentences to get the weight of each sentence.

3) Model *GW* regards words as nodes. Two words are linked by an edge if they co-occur in adjacent three words of any sentences of a paper. Model *GW* first calculates word weights by the Page-Rank algorithm, and then computes a sentence weight by summing the weight of words in that sentence.

4) Model *Context*, *Section*, *SecContext*, *SecTitle* and *GS_Context* take the structural information of a scientific paper into consideration and construct Semantic Link Network instances using the *is-part-of* links among words, sentences, contexts, paragraphs and sections[1]. Besides, the model *GS_Context* combines the model *Context* with the model *GS* by adding *similarity* links among sentences. The sentence weight calculation of these models is an iterative weight updating progress among nodes, in which the word weights

---

[1] Tian J, Cao M, Liu J, Sun X, Zhuge H. Sentence Ranking with the Semantic Link Network in Scientific Paper. Proceedings of the 11th International Conference on Semantics, Knowledge and Grids (SKG) 2015. pp. 73-80.

determine the sentence weights, the sentence weights determine the context weights, the context weights determine the paragraph weights, the paragraph weights determine the section weights, and finally the word weights are in turn updated by sentence, context, paragraph, and section weights.

C. Cause-effect link combining schemes

We proposed four schemes *CE-Filter*, *CE-Pure*, *CE-Bias*, and *CE-Iter* to incorporate *cause-effect* links into each benchmark model. Experiments show the improvements on the quality of summaries when combining the *cause-effect* links with previous models.

1) CE-Filter scheme

The CE-Filter scheme removes the sentences that do not contain *cause-effect* links from the ranking list of sentences produced by a benchmark model, and extracts top-*k* sentences remained in the list as the summary.

Fig 3 illustrates the *CE-Filter* scheme, where $S_1, S_2, \ldots, S_6$ are sentences that compose a paper, CE_1 is the cause-effect link between $S_1$and S_2, CE_2 is contained in S_3, and CE_3 is contained in S_6, and the summary is limited to three sentences. *CE-Filter* removes sentences S_5 and S_4. S_1, S_2, S_3 are kept in the summary.

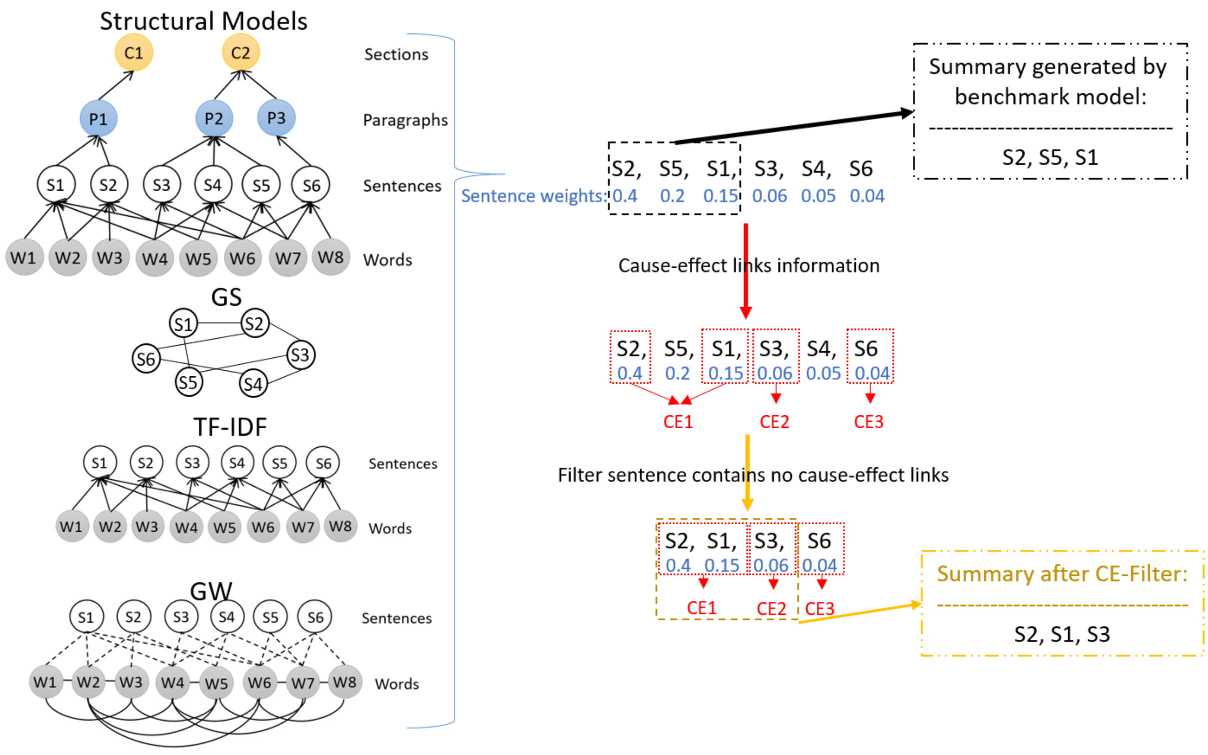

Fig 3. The illustration of *CE-Filter* scheme.

2) *CE-Pure scheme*

The CE-Pure scheme changed the *GS* model by composing a *causal-similarity graph* for a paper. It first removes the non-cause-effect sentences from the *similarity graph* and then adds *cause-effect* links to the remaining *similarity graph*. A Page-Rank algorithm run on the *causal-similarity graph* for ranking sentence nodes, and top-k sentences are selected as a summary.

Fig 4 illustrates the *CE-Pure* model. The construction of a *causal-similarity* graph of a paper is through five steps:

a) Extract sentences that contain *cause-effect* links as the graph's nodes. In Fig 4, S_1, S_2, S_3 and S_6 are extracted as nodes.

b) For each *cause-effect* link that consists of multiple sentences, add an edges (denoted as *causal edge*) between any two sentences that compose this link. In Fig 4, CE_1 consists of two sentences S_1 and S_2. So an undirected edge between S_1 and S_2 is added.

c) For any two nodes, an undirected edge weighted by the *Jaccard* distance is added between them (denoted as *similar edge*). The *Jaccard* distance between sentence S_i and sentence S_j is defined as:

$$\text{Jaccard distance}(S_i, S_j) = \frac{|S_i \cap_{words} S_j|}{|S_i \cup_{words} S_j|}$$

d) Combine a *causal edge* and a *similar edge* between any two nodes by a bias factor *cesim_bias* to form a *causal-similar edge*. We choose *cesim_bias* = 0.333333 on the *OBSERVATION* dataset and the *EMY* dataset by testing the *CE-Pure* model on papers f0001, f0002 and f0003 with the annotated *cause-effect* links.

e) The *causal-similarity graph* is finally normalized in out-degree direction of each node.

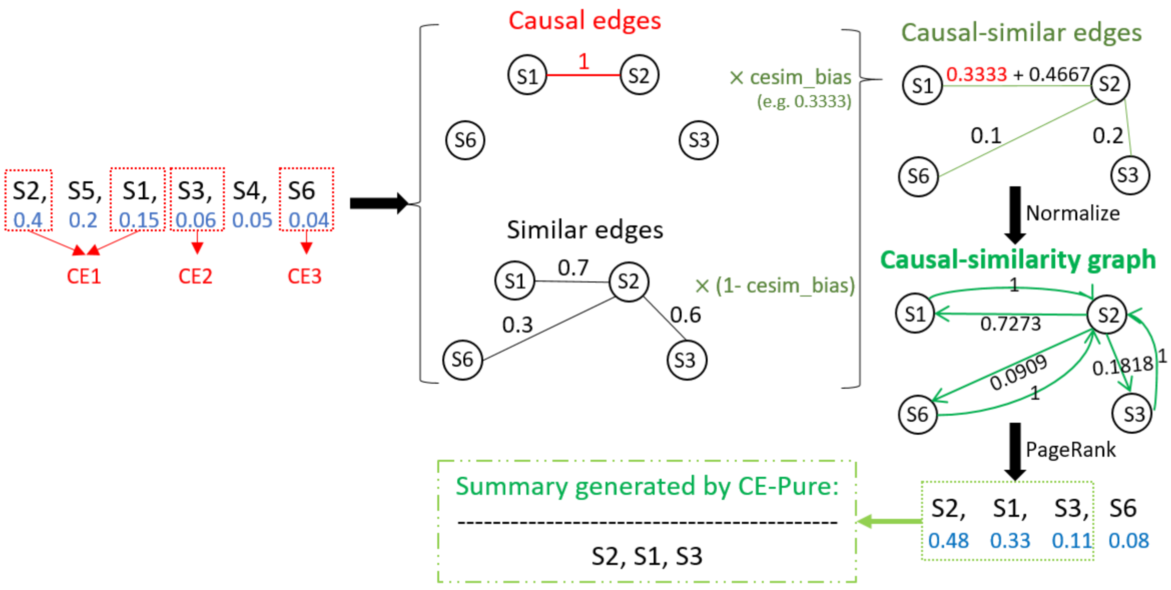

Fig 4. The illustration of *CE-Pure* scheme.

3) CE-Bias scheme

The CE-Bias scheme combines the *CE-Pure* model with previous benchmark models. That is, the weight of a sentence is a weighted summation of the weight computed by the *CE-Pure* model and the weight computed by a benchmark model.

The weighting factor *cebias* controls the balance between the *CE-Pure* model and a benchmark model. We tested *cebias* = 0.05, 0.10, 0.15… 1.0 on papers f0001, f0002 and f0003 with the annotated *cause-effect* links, and choose *cebias* =0.65 on the *OBSERVATION* dataset and the *EMY* dataset.

4) CE-Iter scheme

The *CE-Iter* scheme extends the benchmark model that consists of *is-part-of* links by adding *causal-similarity graph* among sentence nodes (extended models include *Para*, *Context*, *Section*, *SecContext*, *SecTitle* and *GS_Context*).

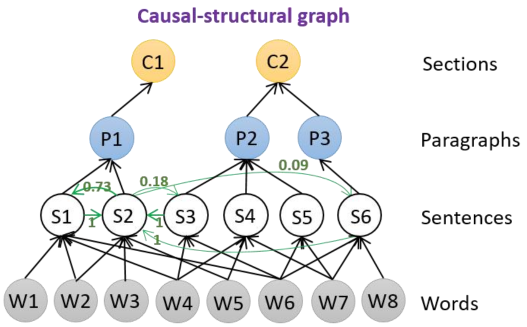

$$s^{(n)} = W\_S^T * \widehat{w}^{(n)} \times (1\text{-}\ ceiter) + CE\_S \times s^{(n\text{-}1)} \times ceiter$$

$$p^{(n)} = S\_P^T * \hat{s}^{(n)}$$

$$c^{(n)} = P\_C^T * \hat{p}^{(n)}$$

$$w^{(n+1)} = W\_S \times \hat{s}^{(n)} + W\_S \times S\_P \times \hat{p}^{(n)} + W\_S \times S\_P \times P\_C \times \hat{c}^{(n)}$$

Fig 5. The illustration of *CE-Iter* scheme.

Fig 5 illustrates the *CE-Iter* scheme. We use a sentence-to-sentence matrix *CE_S* to represent the weights of edges in a *causal-similarity graph*. A sentence weight is computed by combining word weights from the structural link iteration and from the weight obtained by the *causal-similarity graph*. In the formulas, $s^{(n)}$ denotes the sentence weight vector after the n_{th} iteration computed by combing the weights from the word-to-sentence graph *W_S* and

the weights from the *causal-similarity graph CE_S*, $p^{(n)}$ denotes the paragraph weight vector computed by the sentence-to-paragraph graph *S_P*, $c^{(n)}$ denotes the section weight vector computed from paragraph-to-section graph *P_C*, and $w^{(n+1)}$ denotes the word weight vector after $(n+1)_{th}$ iteration computed by weights from sentence, paragraph, and section through the multiplications of their adjacent matrices.

We tried *ceiter* = 0.05, 0.10, 0.15… 1.0 on paper f0001, f0002 and f0003 with annotated *cause-effect* links, and finally choose *ceiter*= 0.65 for *CE-Iter* on the *OBSERVATION* dataset and the *EMY* dataset.

D. Summarization results

Fig 6 shows the ROUGE-1 scores for all models for this experiment. The annotated *cause-effect* links (on the *OBSERVATION* dataset) and the auto-extracted *cause-effect* links (on the *EMY* dataset) are separately tested for generating summaries. Observations are listed below:

1) *The quality of automatically generated summaries is improved by cause-effect links.*

The average ROUGE-1 F-score is improved when combining *cause-effect* links with the original benchmark models. The improvements over six structural benchmark models *Para*, *Context*, *Section*, *SecContext*, *SecTitle* and *GS_Context* are especially prominent. It demonstrates that *cause-effect* links are useful for identifying the important sentences within paper.

2) *The core of papers can be better represented if more types of links are appropriately incorporated.*

The *CE-Pure* model uses *similarity* link and *cause-effect* link to automatically generate summaries for paper. Its *Average F-score* is higher than the *GS* model which just uses the *similar* link. The *CE-Filter* scheme directly imposes the *cause-effect* link on a benchmark model by removing the non-cause-effect sentences. The *CE-Bias* and *CE-Iter* schemes add *cause-effect* link and *similarity* link into a benchmark model by using a *causal-similarity graph* to compute sentence weights. More types of semantic links are used, higher ROUGE-1 scores the models achieve. This demonstrates that a better quality of summarization is achieved with more kinds of semantic links are appropriately combined for constructing the instance model of Semantic Link Network.

3) *Cause-effect links extraction algorithm is effective.*

The ROUGE-1 scores of models combined with the auto-extracted *cause-effect* links as shown in Fig 6(b) are not as high as the models combined with the manually annotated *cause-effect* links as shown in Fig 6(a), but are still better than the original benchmark models that do not use the *cause-effect* links. The poor performance on the *Average Precision* of the ROUGE-1 scores in Fig 6(b) is due to the relatively low precision of the *cause-effect* link extraction algorithm. The *Average F-score* is acceptable because of the high recall rate of the extraction algorithm.

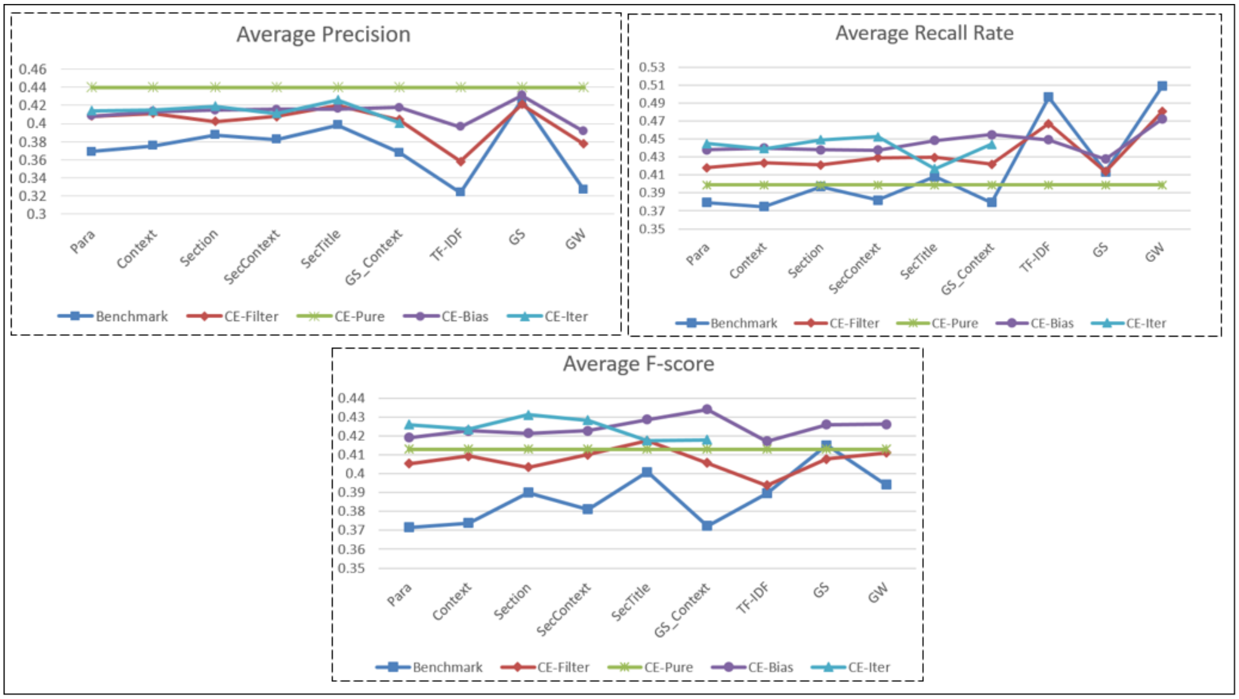

(a) On the *OBSERVATION* dataset using manually annotated cause-effect links

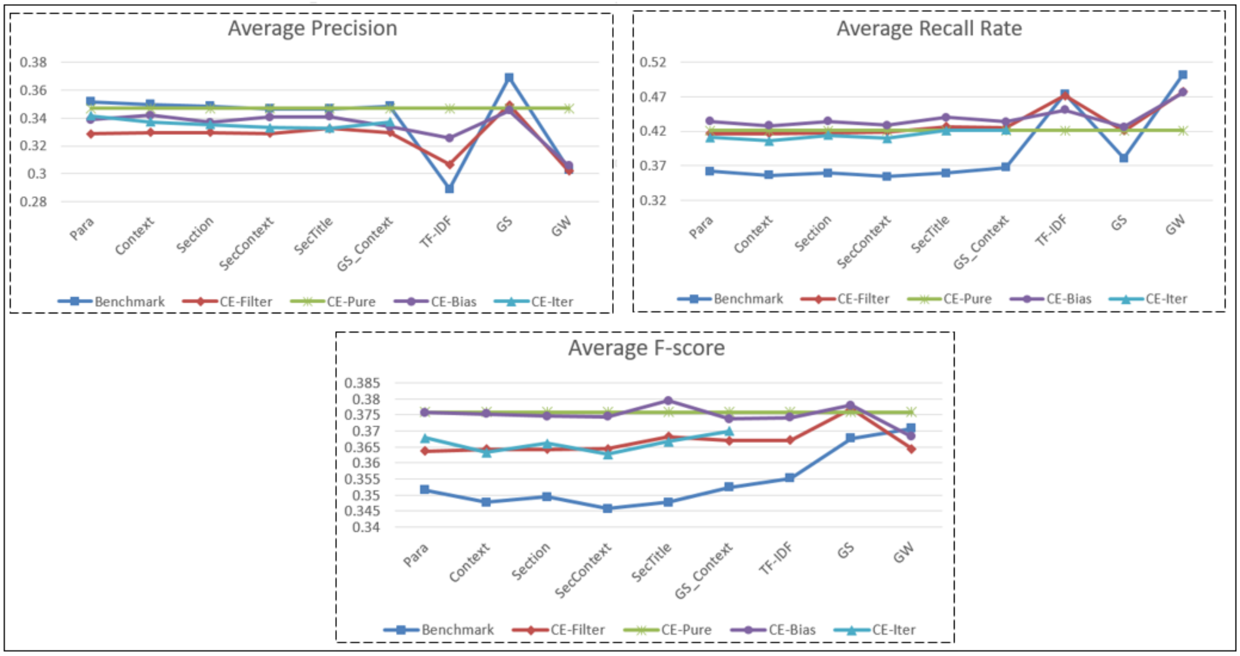

(b) On the *EMY* dataset using automatically extracted cause-effect links

Fig 6. ROUGE-1 scores of nine summarization models of incorporating cause-effect link on the OBSERVATION dataset and EMY dataset.

E. The Impact of the intensity of cause-effect link on summarization quality

Proposition. *The intensity of cause-effect representations is in nonnegative correlation to the quality of the summary of the text they belong to.*

To verify this proposition, we conducted another set of automatic summarization experiment using only manually annotated *cause-effect* links for the papers in the *OBSERVATION* dataset. Six structural benchmark models (i.e., *Para*, *Context*, *Section*, *SecContext*, *SecTitle* and *GS_Context*) incorporating *CE-Bias* and *CE-Iter* schemes were applied to each section to automatically generate summaries for sections.

We invited the annotators who annotated *cause-effect* links as the summarizers to compose standard summaries. For each section of a paper in the *OBSERVATION* dataset, we asked the students to write two standard summaries. One standard summary is written by extracting several original sentences (or clauses) from this section, while the other is composed by integrating original sentences with the students' own understandings on this section. All standard summaries are restricted as shorter than the half-length of the section. The average value of the ROUGE-1 F-scores of six benchmark models is first collected as the basis benchmark score of each section of a paper. Then, applying the *CE-Bias* scheme and the *CE-Iter* scheme on each of six benchmark model, we have another two sets of the ROUGE-1 F-scores for each section of the paper. We compute two average values out of those two sets of F-scores as the final score for each section of a paper for the *CE-Bias* scheme and the *CE-Iter* scheme respectively.

Fig 7 shows the performance of the benchmark models under *CE-Bias*, *CE-Iter* and the six original structural models on each section of nine papers. Each sub-figure corresponds to a paper. The horizontal axis is the section IDs sorted increasingly by their *Annotated Cover Rate* values (the details of *Section ID* and *Annotated Cover Rate* values for each section are presented in Appendix 4). The vertical axis is the average value of the ROUGE-1 scores for each type of models.

The result shows that the sections whose *Annotated Cover Rate* are larger than 16% tend to have automatically generated summaries with a higher average ROUGE-1 score. More than 83.7% of summaries on sections with a larger *Annotated Cover Rate* are scored higher than 0.6 by the tested models. In order to facilitate the observation, we draw a dotted horizontal line at the average ROUGE-1 score of 0.6, and a red vertical dotted line corresponding to the first section whose *Annotated Cover Rate* value is larger than 16%. There are five exceptions of the whole 74 sections, each of which has a low *Annotated Cover Rate* with a high ROUGE-1 score or a high *Annotated Cover Rate* with a low ROUGE-1 score.

So, if the intensity of cause-effect representation of a section reaches a certain level, summaries automatically generated by the instance models of Semantic Link Network for this section are likely to be more satisfactory. It also implies that the intensity of cause-effect representation can be a useful index in judging the difficulty of organizing and representing the core of each section of a scientific paper by semantic links. The verification of this issue on a larger set of more types of texts opens to further studies.

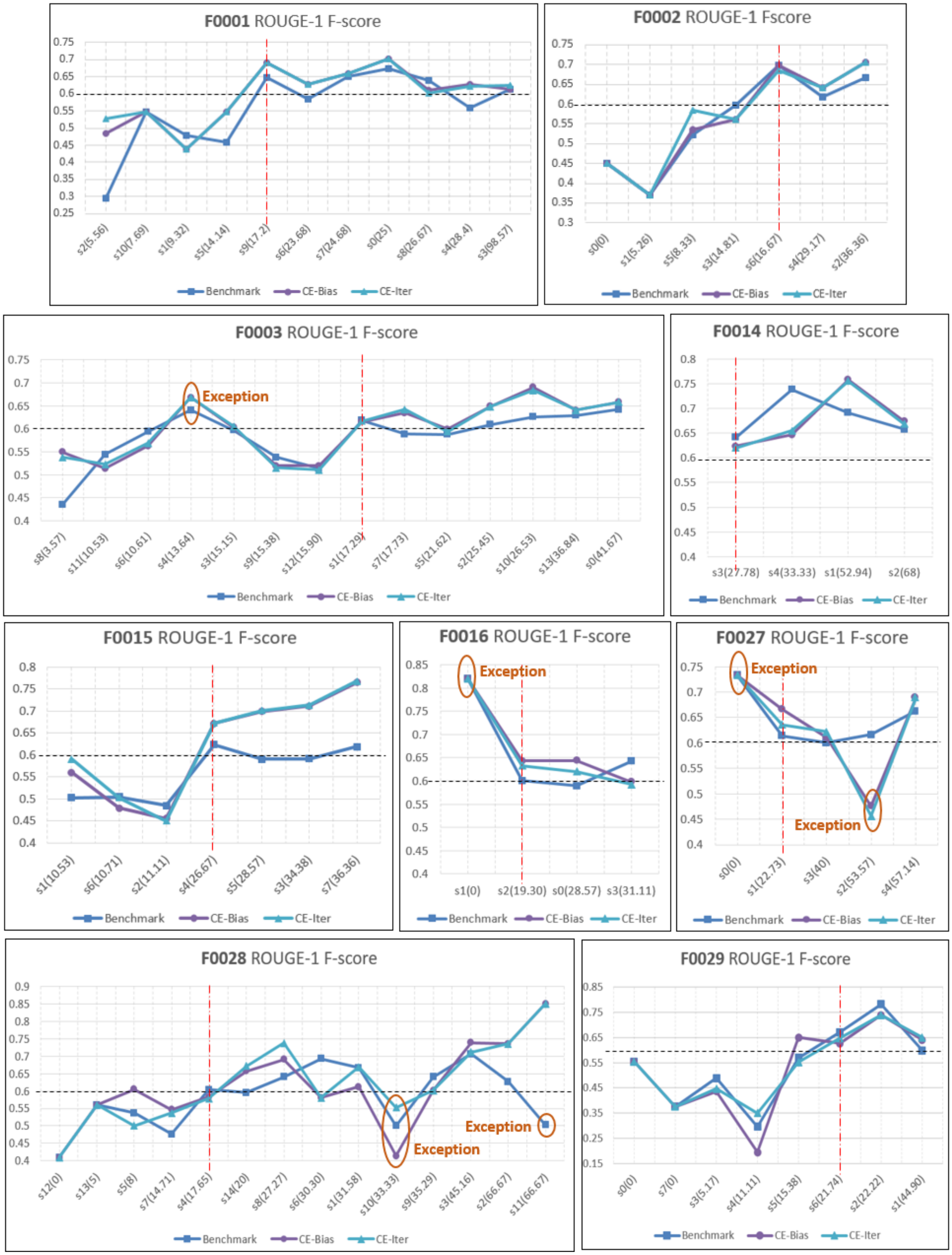

Fig 7. The average ROUGE-1 F-scores of the sections of nine papers on the *OBSERVATION* dataset.
