# Supplementary material for: The contribution of cause-effect link to representing the core of scientific paper—The role of Semantic Link Network
Source: PLoS One. 2018 Jun 21;13(6):e0199303. doi: 10.1371/journal.pone.0199303 (PMC6013162; doi:10.1371/journal.pone.0199303)
Supplement: S7 Appendix — (PDF) [file pone.0199303.s007.pdf]

Appendix 7. Extended experiments on ACL2014 dataset

We extend our experiments to an *ACL2014* dataset that contains 173 conference papers. These papers were downloaded from [the ACL 2014 webpage of the ACL Anthology]. *Cause-effect* links were automatically extracted from each paper for summarization.

A. Proposition 1 on ACL2014 dataset

Table 17 shows the *Extracted Cover Rate* distribution of the extracted *cause-effect* links on the sections of the first ten papers in the *ACL2014* dataset. It is observable that there are some sections with a higher *Extracted Cover Rate* than others, and the sections with a higher *Extracted Cover Rate* have a higher intensity of cause-effect representation in all papers. Proposition 1 holds on the *ACL2014* dataset.

Table 17. The distribution of the extracted *cause-effect* links on the sections of the first ten papers in *ACL2014* dataset.

Article ID	Section ID	Section Title	Sent Num	Extracted Cover Rate
P14-1007	0	Abstract	7	0.0000
	1	Introduction	21	28.5714
	2	Related Work	15	53.3333
	3	System Description	130	39.2308
	4	Experiments	47	25.5319
	5	Discussion and Comparison	19	15.7895
	6	Conclusion and Outlook	12	16.6667
P14-1008	0	Abstract	5	60.0000
	1	Introduction	15	40.0000
	2	The Idea	95	30.5263
	3	Generating On-the-fly Knowledge	50	32.0000
	4	Experiments	53	32.0755
	5	Conclusion and Discussion	24	33.3333
P14-1009	0	Abstract	5	0.0000
	1	Compositional distributional semantics	51	31.3725
	2	The practical lexical function model	56	41.0714
	3	Evaluation	86	34.8837
	4	Conclusion	8	12.5000
	5	Acknowledgements	2	0.0000
P14-1010	0	Abstract	5	20.0000
	1	Introduction	14	50.0000
	2	Related Work	46	19.5652

	3	Methods	109	37.6147
	4	Experimental Setup	42	23.8095
	5	Results	38	21.0526
	6	Conclusion	6	0.0000
P14-1011	0	Abstract	5	0.0000
	1	Introduction	41	43.9024
	2	Related Work	21	28.5714
	3	Bilingually-constrained Recursive Auto-encoders	106	23.5849
	4	Experiments	62	29.0323
	5	Discussions	9	33.3333
	6	Conclusions and Future Work	10	10.0000
p14-1012	0	Abstract	4	50.0000
	1	Introduction	28	53.5714
	2	Related Work	14	28.5714
	3	Input Features for DNN Feature Learning	26	46.1538
	4	Semi-Supervised Deep Auto-encoder Features Learning for SMT	43	39.5349
	5	Experiments and Results	59	3.3898
	6	Conclusions	9	33.3333
P14-1013	0	Abstract	7	28.5714
	1	Introduction	34	38.2353
	2	Background: Deep Learning	10	0.0000
	3	Topic Similarity Model with Neural Network	84	34.5238
	4	Experiments	78	29.4872
	5	Related Work	22	13.6364
	6	Conclusion and Future Work	9	22.2222
P14-1014	0	Abstract	5	0.0000
	1	Introduction	20	40.0000
	2	Learning from Web Text	38	13.1579
	3	Neural Network for POS Disambiguation	35	20.0000
	4	Easy-first POS tagging with Neural Network	31	35.4839
	5	Experiments	71	22.5352
	6	Related Work	37	5.4054
	7	Conclusion	7	0.0000
	8	Acknowledgements	3	0.0000
p14-1015	0	Abstract	8	50.0000
	1	Introduction	31	41.9355
	2	Related Work	15	26.6667
	3	Problem Definition	7	14.2857
	4	Our Approach	96	42.7083
	5	Experimental Evaluation	46	50.0000
	6	Conclusions and Future Work	8	37.5000
p14-1018	0	Abstract	4	50.0000
	1	Introduction	14	28.5714
	2	Identifying Twitter Social Graph	30	33.3333
	3	Batch Models	26	30.7692
	4	Streaming Models	10	30.0000
	5	Experimental Setup	34	5.8824
	6	Static Classification Results	24	33.3333
	7	Streaming Classification Results	48	16.6667
	8	Related Work	33	24.2424
	9	Conclusions and Future Work	26	34.6154

B. Proposition 2 on ACL2014 dataset

Table 18 shows that the extracted *cause-effect* links cover more than 67.9% of the key words on average. So, Proposition 2 also holds on the *ACL2014* dataset.

Table 18. The coverage of key words on the extracted *cause-effect* links on *ACL2014* dataset.

Article ID	Abstract (%)	Conclusion (%)	Abs&Conc (%)
P14-1007	61.6279	54.2105	56.5217
P14-1008	82.5472	74.3494	77.9626
P14-1009	66.6667	60.3053	62.234
P14-1010	77.027	59.2593	67.7419
P14-1011	87.1429	75.9259	80.3371
P14-1012	82.0513	71.519	75
P14-1013	49.1071	80.5825	64.186
P14-1014	69.4915	64.7887	66.9231
P14-1015	91.954	89.6825	90.6103
P14-1018	96.2025	78.0186	81.592
P14-1019	79.7753	69.697	77.0492
P14-1020	63.5417	45.3125	56.25
P14-1022	75.2381	92.4242	81.8713
P14-1023	81.6327	68.1063	70
P14-1024	80.3922	81.9277	81.3433
P14-1025	86.6667	0	86.6667
P14-1027	80.6122	87.907	85.623
P14-1028	81.7073	82.3529	81.9549
P14-1029	70.1299	59.2105	64.7059
P14-1031	77.7778	81.6667	79.4326
P14-1039	95.122	74.5098	81.7021
P14-1043	86.3248	82.6087	84.689
P14-1044	89.6552	81.3333	84.9624
P14-1045	73.1707	82.906	78.8945
P14-1047	83.5443	78.8462	79.941
P14-1048	79.7297	75.4902	77.2727
P14-1050	54.5455	70.5882	63.5762
P14-1052	92.9825	69.0909	77.2455
P14-1054	85.5422	61.5385	73.913
P14-1056	65.3061	82.2222	73.4043
P14-1057	85.1852	86.7925	86.0963
P14-1058	81.5385	79.3103	80.4878
P14-1060	63.5135	70.8333	68.595
P14-1062	62.1951	72.7273	65.2174
P14-1063	92.6471	75.4717	82.1839
P14-1064	74.359	59.5238	69.1667
P14-1065	87.7551	79.0541	81.2183
P14-1067	58.9041	84.8276	76.1468
P14-1068	77.5	68.8889	71.5385
P14-1070	86.5385	77.2727	81.3559
P14-1073	71.2121	37.5	58.4906
P14-1077	87.3239	87.5	87.4172
P14-1079	77.6596	84.0426	80.8511
P14-1082	67.8571	60.3846	62.2093
P14-1083	83.3333	74.6835	78.4173
P14-1085	55	65.1163	60.9589
P14-1087	76.7123	89.4737	83.9286

P14-1088	73.0337	97.2826	89.3773
P14-1090	88	42.0455	58.6957
P14-1091	88.172	100	93.8202
P14-1092	85.7143	66.6667	76.129
P14-1093	90.6977	89.1892	90.2439
P14-1096	81.6514	76.4706	78.3784
P14-1097	91.0448	89.4737	90
P14-1100	88.2353	41.0256	71.028
P14-1101	77.9412	71.4286	73.1061
P14-1102	100	0	100
P14-1103	91.4286	46.3415	74.7748
P14-1104	81.5385	76.25	78.6207
P14-1105	73.7705	55	66.3366
P14-1106	97.1429	79.7619	87.6623
P14-1107	64.9123	84.6154	76.2963
P14-1108	83.7209	94.8718	89.0244
P14-1109	78.7234	83.908	81.2155
P14-1113	76.9231	82.4742	80.2469
P14-1115	85.6164	73.8739	80.5447
P14-1116	85.7143	88.7417	87.7193
P14-1117	69.0141	76.4706	71.4286
P14-1118	75.9259	76.2887	76.1589
P14-1119	48.3871	56.6265	54.386
P14-1121	91.3793	94.6237	93.3775
P14-1122	75	68.0328	70.1149
P14-1123	67.2414	86.0465	78.4722
P14-1124	86.3014	61.8421	73.8255
P14-1126	91.7808	97.9167	94.2149
P14-1128	73.9726	86.5385	79.2
P14-1129	89.7959	0	89.7959
P14-1130	75	80.2326	77.7778
P14-1131	81.8182	85.7143	83.6538
P14-1132	92.2078	68.1529	76.0684
P14-1133	69.5122	0	69.5122
P14-1136	74.6032	63.6364	68.9922
P14-1138	84.8101	78.8235	81.7073
P14-1140	78.3133	79.7753	79.0698
P14-1142	61.5385	73.6842	66.6667
P14-1144	73.0769	79.0698	75.7895
P14-1145	83.0189	88.8889	86.2069
P14-1146	91.8919	97.8495	94.6078
P14-2004	77.551	78.5714	78.022
P14-2005	90.6977	85.4167	87.9121
P14-2007	65.0602	73.1343	68.6667
P14-2008	75.6098	77.7778	76.4706
P14-2010	80.3571	52	62.1795
P14-2012	79.1667	0	79.1667
P14-2013	77.1429	89.1892	81.3084
P14-2016	87.8788	67.4419	76.3158
P14-2017	87.037	81.6092	82.8947
P14-2019	43.1818	65.8228	57.7236
P14-2026	85.4167	91.3043	88.8889
P14-2028	51.3514	62.963	59.322
P14-2029	45.098	74.7475	64.6667
P14-2030	65.5172	75	70.8955
P14-2031	60.8696	55.4054	57.5
P14-2032	54.2373	54.902	54.5455
P14-2034	31.0811	48.3871	38.9706
P14-2035	100	85	92.5
P14-2036	96.0784	91.2281	93.5185
P14-2039	38.7097	75.5556	54.2056

P14-2040	95.2381	85	92.7711
P14-2044	79.6296	46.0317	61.5385
P14-2045	78.2609	89.5652	85.3261
P14-2046	62.069	65.9574	64.4737
P14-2047	81.9672	74.2424	77.9528
P14-2048	95.2381	0	95.2381
P14-2049	65.8537	37.5	53.4247
P14-2050	60	56.9231	58.0952
P14-2051	88.4615	64.3836	74.4
P14-2052	92.5373	86.4078	88.8235
P14-2053	63.4921	70.3297	67.5325
P14-2054	64.5161	69.4444	67.1642
P14-2055	91.6667	61.1111	77.193
P14-2058	66.6667	93.75	84
P14-2059	96.4912	97.2222	96.9697
P14-2060	69.2308	69.2308	69.2308
P14-2061	70.1493	33.3333	49.3506
P14-2062	67.3913	56.8182	62.2222
P14-2063	54.7619	57.377	55.8621
P14-2065	35	79.7297	59.7015
P14-2066	81.3559	0	81.3559
P14-2068	67.7419	60.3175	64
P14-2069	35.3535	85.1351	56.6474
P14-2070	90.625	87.0588	88.5906
P14-2071	69.0909	46.1538	59.5745
P14-2073	61.9048	64.1791	63.3028
P14-2074	52.3256	66.6667	59.7765
P14-2078	94.8718	75.8065	83.1683
P14-2080	63.0435	72.549	68.0412
P14-2082	68.9655	64.4444	67.4242
P14-2083	45.614	35.8974	41.6667
P14-2084	94.3396	70.7317	80
P14-2085	78.3333	53.6842	63.2258
P14-2087	70	70.5882	70.4
P14-2088	62.7451	69.2308	66.0194
P14-2089	72.7273	76.6234	75.2066
P14-2092	59.5238	60.3774	60
P14-2095	72.7273	53.3333	64
P14-2096	70.2703	76.7677	73.9884
P14-2098	58.5366	45.8333	51.6854
P14-2099	61.5385	69.7674	65.8537
P14-2101	74.2574	79.5455	75.8621
P14-2103	63.0435	68.2927	65.5172
P14-2104	84.7458	62.8571	76.5957
P14-2105	80.597	63.1068	70
P14-2106	60.7843	68.6567	65.2542
P14-2108	65.1163	64.3836	64.6552
P14-2109	71.4286	0	71.4286
P14-2110	68.5185	0	68.5185
P14-2111	72.0588	68.8525	70.5426
P14-2113	63.2653	37.037	46.9231
P14-2114	78.5714	69.4118	73.5484
P14-2118	50	74.0741	70.5263
P14-2119	52.0833	79.0698	64.8352
P14-2120	73.3333	86	83.0769
P14-2121	72.8814	0	72.8814
P14-2122	83.5443	75.7282	79.1209
P14-2127	95.3846	56	84.4444
P14-2128	79.6296	64.8148	72.2222
P14-2129	76.1194	51.5385	59.8985
P14-2133	80.303	84.4262	82.9787

P14-2134	60.6061	68.5393	65.1613
P14-2135	69.2308	73.0769	71.7949
P14-2137	79.5918	72.4138	76.9231
P14-2138	76.9231	70.9402	72.7811
**Average**	**75.08287**	**67.93724**	**73.86413**

C. **Impact of cause-effect links on automatic summarization**

Nine benchmark models and four schemes of combining *cause-effect* links with other semantic links introduced in Appendix 6 are tested on the ACL2014 dataset. We set *cesim_bias* = 0.16, *cebias* = 0.43, *ceiter* = 0.86 on the *ACL2014* dataset for combining the extracted *cause-effect* links with other semantic links. The values of *cesim_bias* and *cebias* are lower than those used in the summarization experiments on the *EMY* dataset. These lower bias factors in *CE-Pure* and *CE-Bias* weaken the weight transfer between sentences that do not have *cause-effect* links. The value of *ceiter* is higher than those used in the summarization experiments on the *EMY* dataset. High *ceiter* expands the impacts of the correctly extracted *cause-effect* links when applying the *CE-Iter* scheme to the six structural benchmark models.

Fig 8 shows the performance of each benchmark model under different schemes of combing semantic links:

1) *The quality of automatically generated summaries is improved after using cause-effect link*.

The average-F scores of most benchmark models are improved when combining with the extracted *cause-effect* links by schemes *CE-Filter* and *CE-Bias*. Improvements over the six structural benchmark models are significant. The performance of the *CE-Pure* scheme is a bit worse than the five of the structural benchmark models, and the performance of the *CE-Iter* scheme is almost the same as the benchmark models. This may due to the incorrect weight

transfer between sentences that have no *cause-effect* link. However, the *CE-Pure* scheme is better than *TF-IDF, GS* and *GW* models, indicating that the *cause-effect* link is still helpful for facilitating the Semantic Link Network to identifying important sentences.

2) *The core of papers can be better represented if more types of links are appropriately incorporated*.

The *CE-Pure* model builds a c*ausal-similarity* graph for each paper with the *cause-effect* link and the *similar* link. Its Average F-score is higher than that of *GS* model (which just uses the *similar* link), *TF-IDF model, and GW* models (which just use *is-part-of* link between words and sentences) on the *ACL2014* dataset. The *CE-Filter, CE-Bias* and *CE-Iter* combine *cause-effect* link into each original benchmark models, and they achieved better performances on the *ACL2014* datasets by taking more types of semantic links than each benchmark model.

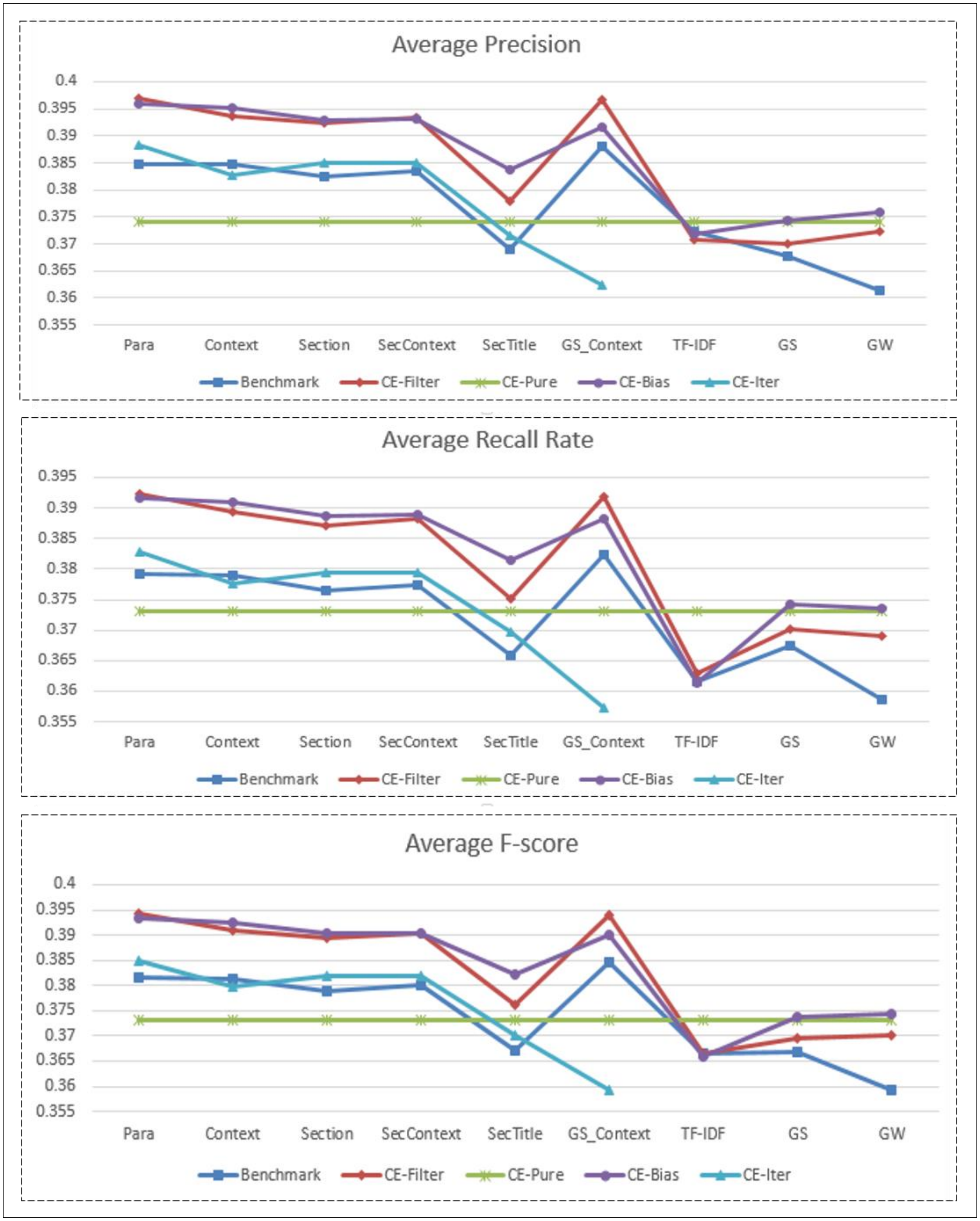

Fig 8. The ROUGE-1 scores of nine summarization models of incorporating cause-effect link on *ACL2014* dataset.
