# Supplementary material for: The contribution of cause-effect link to representing the core of scientific paper—The role of Semantic Link Network
Source: PLoS One. 2018 Jun 21;13(6):e0199303. doi: 10.1371/journal.pone.0199303 (PMC6013162; doi:10.1371/journal.pone.0199303)
Supplement: S8 Appendix — (PDF) [file pone.0199303.s008.pdf]

APPENDIX 8. Details of the EMY dataset

Table 19 lists the papers of the *EMY* dataset, which were selected according to the diversity of the types of papers and the familiarity of the annotators.

Table 19. Papers in the *EMY* dataset.

ID	Title	Source
f0001	Dimensionality on Summarization	arXiv:1507.00209, 2015.
f0002	Discovery of Knowledge Flow in Science	Communications of the ACM, 2006, 49(5): 101-107.
f0003	Semantic Linking Through Spaces for Cyber-Physical-Socio Intelligence: A Methodology	Artificial Intelligence, 2011, 175(5-6): 988-1019.
f0004	Semantics, Knowledge and Grids	Concurrency and Computation: Practice and Experience, 2015, 27(15): 3912-3914.
f0005	Mapping Big Data into Knowledge Space with Cognitive Cyber-Infrastructure	arXiv:1507.06500, 2015.
f0006	Cyber-Physical Society: the Science and Engineering for Future Society	Future Generation Computer Systems, 2014, 32(C): 180-186.
f0007	Faceted Search, Social Networking and Interactive Semantics	World Wide Web, 2014, 17(4): 589.
f0008	Probabilistic Resource Space Model for Managing Resources in Cyber-Physical Society	IEEE Transactions on Services Computing, 2012, 5(3): 404-421.
f0009	Basic Operations, Completeness and Dynamicity of Cyber Physical Socio Semantic Link Network CPSocio-SLN	Concurrency and Computation: Practice and Experience, 2011, 23(9): 924-939.
f0010	Automatically Constructing Semantic Link Network on Documents	Concurrency and Computation: Practice and Experience, 2011, 23(9): 956-971.
f0011	Interactive Semantics	Artificial Intelligence, 2010, 174(2): 190-204.
f0012	Special Section: Semantic Link Network	Future Generation Computer Systems, 2010, 26(3): 359-360.
f0013	The Schema Theory for Semantic Link Network	Future Generation Computer Systems, 2010, 26(3): 408-420.
f0014	The Inversion of Functions Defined by Turing Machines	C E Shannon & J Mccarthy Automata Studies, Annals of Mathematical Studies 1970, 35(3): 481-481.
f0015	The Little Thoughts of Thinking Machines	Psychology Today, 1983, 17(12): 46-49.
f0016	Some Expert System Need Common Sense	Annals of the New York Academy of Sciences, 1984, 426(1): 129-137.
f0017	Programs with Common Sense	Semantic Information Processing, 1959, 130 (5): 403-418.
f0018	Epistemological Problems of Artificial Intelligence	Readings in artificial intelligence, 1981: 459-465.
f0019	What Has AI in Common with Philosophy?	IJCAI, 1995: 2041-2044.
f0020	Approximate Objects and Approximate Theories	Proceedings of 7th International Conference on Principles of Knowledge Representation and Reasoning, 2000: 519-526.
f0021	Coloring Maps and the Kowalski Doctrine	A Stanford report, 1982. See: https://web.archive.org/web/20131004215729/http://www-formal.stanford.edu/jmc/coloring.pdf
f0022	Creative Solutions to Problems	AISB'99 Symposium on AI and Scientific Creativity, 1999: 44-48.
f0023	Simple Deterministic Free Will	Unpublished note, 2005. See: https://web.archive.org/web/20131004215919/http://www-formal.stanford.edu/jmc/freewill2.pdf
f0024	Generality in Artificial Intelligence	Communications of the ACM, 1987, 30(12): 1030-1035.

f0025	A logical AI Approach to Context	Unpublished note, 1996. See: https://web.archive.org/web/20131004215656/http://www-formal.stanford.edu/jmc/logical.pdf
f0026	Todd Moody's Zombies	Journal of Consciousness Studies, 1995, 2(4): 345-347.
f0027	2020 Computing: Science in an Exponential World	Nature, 2006, 440(7083): 413
f0028	Scientific Data Management in the Coming Decade	ACM Sigmod Record, 2005, 34(4): 34-41.
f0029	Cross-Matching Multiple Spatial Observations and Dealing with Missing Data	arXiv:cs/0701172, 2007.
f0030	Scientific data federation: The World Wide Telescope	Chapter 7 in *The Grid 2* (2), 2004, pp.95-108.
f0031	The World-Wide Telescope	Science, 2001, 293(5537): 2037-2040.
f0032	A "Measure of Transaction Processing" 20 Years Later	arXiv:cs/0701162, 2007.
f0033	Thousands of Debit Credit Transactions-Per-Second: Easy and Inexpensive	arXiv:cs/0701161, 2007.
f0034	Where the Rubber Meets the Sky: Bridging the Gap between Databases and Science	arXiv:cs/0502011, 2005.
f0035	The World-Wide Telescope: An Archetype for Online Science	CACM, 2002, 45(11): 50-54.
f0036	Rules of Thumb in Data Engineering	Proceedings of 16th International Conference on Data Engineering, IEEE, 2000, 42(2): 3-10.
f0037	Functionality, Availability, Agility, Manageability, Scalability -- The New Priorities of Application Design	Proc. Int'l Workshop High Performance Trans. Systems. 2001: 1-6.
f0038	TeraScale SneakerNet: Using Inexpensive Disks for Backup, Archiving, and Data Exchange	arXiv:cs/0208011, 2002.
f0039	Computer Technology Forecast	Virtual Observatories of the Future, 2001, vol.225, pp.241. See: https://www.microsoft.com/en-us/research/wp-content/uploads/2016/02/tr-2000-102.pdf
